# Assessing Women’s Negative Sanitation Experiences and Concerns: The Development of a Novel Sanitation Insecurity Measure

**DOI:** 10.3390/ijerph14070755

**Published:** 2017-07-11

**Authors:** Bethany A. Caruso, Thomas Clasen, Kathryn M. Yount, Hannah L. F. Cooper, Craig Hadley, Regine Haardörfer

**Affiliations:** 1 Department of Behavioral Sciences and Health Education, Rollins School of Public Health, Emory University, Atlanta, GA 30322, USA; hcoope3@emory.edu (H.L.F.C.); regine.haardoerfer@emory.edu (R.H.); 2 Department of Environmental Health, Rollins School of Public Health, Emory University, Atlanta, GA 30322, USA; thomas.f.clasen@emory.edu; 3 Hubert Department of Global Health, Rollins School of Public Health, Emory University, Atlanta, GA 30322, USA; kathryn.yount@emory.edu; 4 Department of Sociology, Emory University, Atlanta, GA 30322, USA; 5 Department of Anthropology, Emory University, Atlanta, GA 30322, USA; chadley@emory.edu

**Keywords:** sanitation, water, gender, defecation, urination, security, factor analysis, differential item functioning

## Abstract

Lack of access to acceptable sanitation facilities can expose individuals, particularly women, to physical, social, and mental health risks. While some of the challenges have been documented, standard metrics are needed to determine the extent to which women have urination- and defecation-related concerns and negative experiences. Such metrics also are needed to assess the effectiveness of interventions to mitigate them. We developed a sanitation insecurity measure to capture the range and frequency of women’s sanitation-related concerns and negative experiences. Research was conducted in rural Odisha, India with women across various life course stages to reflect a range of perspectives. This paper documents the mixed data collection methods and the exploratory and confirmatory factor analyses we employed to arrive at a final measure. The final sanitation insecurity measure includes 50 items across seven factors that reflect the physical environment, the social environment, and individual-level constraints. Most factor scores were significantly higher for unmarried women and for women who lacked access to functional latrines, indicating social and environmental influence on experiences. This measure will enable researchers to evaluate how sanitation insecurity affects health and to determine if and how sanitation interventions ameliorate women’s concerns and negative experiences associated with sanitation.

## 1. Introduction

Research to date on sanitation and health has focused on links between exposure to pathogens and risk of infectious diseases (diarrhea, soil-transmitted helminthes, and trachoma) or the longer term impacts of these infectious diseases, particularly among children under five (tropical enteropathy, stunting, and cognitive impairment) [1,2,3,4,5,6,7,8]. Assessing health impacts related to pathogen exposures and their downstream effects alone may not capture the breadth of negative health outcomes that may be associated with compromised water, sanitation, and hygiene environments; individuals’ experiences attending to their sanitation needs may be harmful to health [9]. Yet, there is no current means to assess quantitatively the presence and intensity of negative sanitation experiences. The purpose of this paper is to document the development of a novel measure to assess Sanitation Insecurity, a construct capturing concerns and negative experiences related to sanitation.

A poor sanitation environment typically has been considered to be one where a toilet is non-existent or is “unimproved”—that is, incapable of hygienically separating human excreta from human contact. An estimated 2.4 billion people lack access to improved sanitation, with 946 million of those people practicing open defecation because they lack access to any form of sanitation facility whatsoever [10]. While the number of people without access to facilities is staggering, current estimates of improved sanitation coverage do not consider privacy, cleanliness, safety, comfort, accessibility, or acceptability of facilities from the perspective of those who have them. Nor do estimates consider use. Ownership of a household latrine does not equate to use, which is well documented in India. In a cross-sectional study in Orissa, over a third of households with a latrine did not have a single household member using it [11]. In another study across six Indian states with 3235 households representing over 22,000 individuals, Coffey et al. found that 48% of households that had a latrine had at least one family member that did not use it [12]. As such, even those with “improved” facilities may have poor sanitation environments that could compromise health outcomes.

Research has revealed that the experience of urinating, defecating, and managing menstruation can pose challenges that may expose individuals, particularly women, to physical, social, and mental health risks. For example, in India, the practice of open defecation has been associated with adverse pregnancy outcomes and pre-term birth [13]. In Kenya and India, women practicing open defecation have higher odds of experiencing non-partner violence [14,15]. Poor sanitation conditions, even for people with improved facilities, may pose threats to women’s safety and dignity. Women have reported that poor sanitation environs have caused disgust due to filth, interfered with responsibilities because of long lines or distant locations for sanitation needs, failed to accommodate cultural practices or sex-specific needs, reinforced women’s lack of control over their environment, and caused feelings of marginalization and negative identity [16,17,18,19,20,21]. Moreover, an emerging line of research has qualitatively identified sanitation as a cause of stress and anxiety, particularly among women [21,22,23,24,25].

Inspired by research that has investigated and created measures for culturally grounded constructs of food and water insecurity [26,27,28,29,30,31,32,33,34], the aim of this paper is to document the development of a novel measure for sanitation insecurity, with a specific focus on women. Food and water insecurity have been defined as insufficient and uncertain access to adequate food and water for an active lifestyle [33,35]. As Hadley and Wutich (2009) note, these definitions are multidimensional and represent not only the biological needs people have associated with food and water, but also the social needs [33]. Adequacy is typically assessed with indicators of intake, like calories consumed or water used. Access can be assessed by evaluating availability of water sources or food markets, time to sources, and associated costs among others. However, measures of access and adequacy alone may under-estimate how insecurity impacts health and well-being [33]. The lifestyle dimension of the definition considers the culture, experiences, and perceptions associated with water and food needs, which are primarily social in nature. These may include, for example, food preferences and norms around water use for hygiene and cleanliness [33,36]. To account for the lifestyle dimension beyond typical assessments of access and adequacy alone, researchers investigating food and water insecurity fields have developed measures that reflect the *experience* of insecurity [30,33,34]. This approach allows for consideration of the socio-cultural context that may influence how individuals experience insecurity [34]. Insecurity measures accounting for experiences have demonstrated associations with psychosocial distress [32,34].

The concept of “toilet insecurity” has been discussed as “when safe, usable toilets are not available” [37]. This definition focuses on access (availability) and specific elements of adequacy (safety, usability). As with research on food and water, the growing body of qualitative research on sanitation suggests that the socio-cultural context influences how individuals experience sanitation. More specifically, socio-cultural interpretations of gender and associated gender roles and expectations influences how women experience sanitation. Kandiyoti, a gender scholar, argues that women operate within a gendered context and that this gendered context has “concrete constraints” that effect or dictate what strategies women have available to optimize their life choices [38]. Considering sanitation, research has documented a range of strategies that women have had to adopt in order to meet their basic sanitation needs due to various constraints on their mobility and agency, like defecating late at night or early in the morning to maintain privacy and dignity, withholding food and water to prevent urination and defecation, and suppressing urges to tend to household obligations or dependent care [22,39,40].

An assessment of sanitation insecurity that is inclusive of women’s experiences and perceptions may better demonstrate how sanitation influences health and well-being above and beyond evaluations of facility access alone. Qualitative research has begun to document the experiences women have when trying to address their sanitation-related needs. Yet, no measure of sanitation insecurity exists to quantify the extent to which women have sanitation-related concerns and negative experiences, or how frequently these concerns or experiences occur. A contextualized measure of sanitation insecurity is needed to understand more fully the range of women’s experiences relating to sanitation and to quantify the extent and frequency of sanitation-related concerns and negative experiences within populations. This measure will enable researchers to assess determinants of sanitation insecurity, understand how sanitation insecurity affects health, including stress and quality of life among other outcomes, and, as has been done with water insecurity tools [41], evaluate if and how interventions effectively ameliorate concerns and negative experiences. As such, this measure of sanitation insecurity also has the potential to be a critical tool for practitioners aiming to deliver sanitation programs that meet the specific needs of women.

## 2. Materials and Methods

### 2.1. Setting

The present research uses the ground-up approaches from food and water insecurity research to create a measure of sanitation insecurity from the perspective of women in rural Odisha, India. A measure of sanitation insecurity is particularly relevant to the Indian context, where only 44% of the total population has access to a sanitation facility and 61% of rural inhabitants practice open defecation [10].

Data were collected from March 2014–February 2015 in rural communities of Puri district, Odisha, India that had been engaged in a cluster randomized controlled trial (CRT) assessing the health impacts of a sanitation intervention (latrine provision) as part of the government funded Total Sanitation Campaign (See Boisson et al. for more detailed information about the intervention) [42,43,44,45]. Over the course of the trial (May 2010–December 2013), mean sanitation coverage increased from 9% to 63% in intervention communities and from 8% to 12% in control communities; no reduction in diarrhea, soil-transmitted helminth infection, or child malnutrition was detected as a result of the intervention [43].

In rural Puri, 94% of households have an improved drinking water source, 37% of households have an improved sanitation facility, 83% of women are literate, 8% of 20–24 year olds reported being married before age 18, 17% of children under age 5 are stunted, 11% are wasted, and 18% are underweight [46]. Most residents are Hindu (97%); 2.7% are Muslim [47]. While there are tribal communities in Odisha, we did not work with these communities in this study.

### 2.2. Overview of Research Design

**Figure 1:**
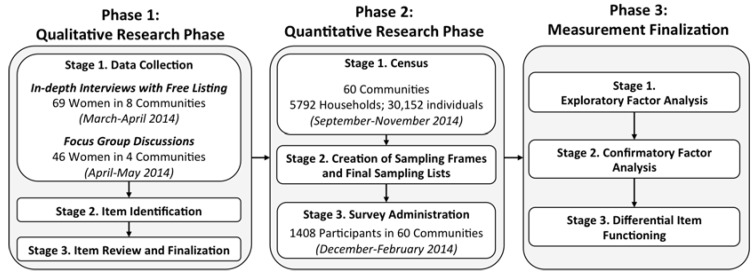
Schematic of Exploratory Sequential Mixed Methods Research Design followed to create Sanitation Insecurity measure.

Researchers who have created experienced-based measures of food and water insecurity started first with qualitative research then used findings to generate surveys and produce scores [30,33,34]. Informed by this process, we followed a sequential mixed methods design with three phases to create and evaluate a measure of sanitation insecurity [48]. An overview of the three phases is depicted in Figure 1 and explained in detail in the text that follows.

During phase one, the qualitative phase, we conducted research to generate items for the measure. During phase two, the quantitative phase, we conducted a census of eligible communities to create a sampling frame and administered a survey with the items to a probability-based sample of women in those communities. During phase three, the measurement finalization phase, we explored the factor structure of the sanitation insecurity items using exploratory factor analysis (EFA), tested the factor structure identified in the EFA using confirmatory factor analysis (CFA), and assessed for measurement non-invariance, or differential item functioning (DIF), of specific scale items using multiple indicator multiple causes (MIMIC) models [49]. From these analyses, we recommend a final set of items for the sanitation insecurity measure and report mean scores for the population engaged.

To create a measure that is applicable to women across various life stages, we only include urination and defecation-related items in this measure. Menstruation is a critical sanitation-related behavior. However, women who are pregnant, have recently given birth, or are of advanced age do not experience menstruation and menstruation-related items would not be applicable to them. Recognizing the need to consider also women’s menstruation-related experiences and concerns, we are developing a separate measure of menstruation insecurity that can be used in tandem when appropriate.

### 2.3. Phase 1: Qualitative Research

The qualitative research phase involved three stages: data collection, item identification, and item review and finalization.

#### 2.3.1. Phase 1, Stage 1: Data Collection

First, we conducted Free-listing interviews (FLIs) to identify items for the sanitation insecurity measure (March–April 2014). Free-listing is an activity used to identify commonly shared perceptions about a topic or concept from a homogenous group of participants [50]. Women were asked to list their concerns related to urination and defecation. For each behavior, we probed about additional concerns they may have at night, during the monsoon season, and about dependents. We interviewed 69 women one-on-one from eight communities (five former intervention, three former control), which were purposively selected to represent varied sanitation coverage and geographical diversity. Women were selected purposively within each community to represent unique life stages: (1) unmarried (*N* = 16); (2) married three years or less (*N* = 12); (3) married over three years (*N* = 22); (4) and women over 49 years of age (*N* = 19).

Second, we conducted eight Focus Group Discussions (FGDs) with 46 women in four different, purposively selected communities (two former intervention and two former control) to gain further detail about concerns expressed in individual interviews (April–May 2014). As with the FLIs, we asked FGD participants to indicate concerns related to urination and defecation. We also inquired about specific concerns mentioned in FLIs about which we sought more detail, and probed about the severity of concerns noted. Four FGDs were held with unmarried women (*N* = 23) and four FGDs with married women (*N* = 23). Trained research assistants facilitated FLIs and FGDs in Oriya, recorded, transcribed, and translated into English. Additional information about the qualitative methods used and in-depth analysis of qualitative findings are reported elsewhere [51].

#### 2.3.2. Phase 1, Stage 2: Item Identification

To generate potential items for inclusion in the measure, we first analyzed the FLIs to understand the scope and frequency of concerns. Of the 69 women who participated in FLIs, 63 indicated having concerns related to urination (29 unique concerns) and 65 indicated having concerns related to defecation (29 unique concerns). FGDs corroborated the concerns noted in FLIs, but provided more detail and context. Overall, concerns were related to three broad domains: the physical environment, the social environment, and individual-level personal constraints.

We created an initial list of items from the FLI and FGD concerns and subsequently omitted all items directly related to the monsoon season because survey administration would not take place during that time to make those questions relevant.

#### 2.3.3. Phase 1, Stage 3: Item Review and Finalization

During this stage, four rounds of item review took place to assess content validity, face validity, and translation. First, draft items were sent to two peer-reviewers with experience researching women’s sanitation in India to assess content validity [52]. The peer-reviewers provided recommendations for revising the wording of items, specifically those related to concerns about harm. Second, to further assess content validity the two research assistants (RAs) who carried out the qualitative data collection reviewed the items and provided comments, with particular attention to alternative phrasing for existing items to be more specific (for example, they suggested asking about a concern for infection as opposed to a concern about health in general). The two RAs then translated the items from English to Oriya independently and then compared translations to reconcile any discrepancies and create a single translation.

Third, the RAs reviewed each translated item with the nine Oriya-speaking female enumerators hired to administer the survey to assess face validity [52]. The enumerators are from the region where the data collection was to take place and had experience with sanitation-related surveys from previous research. They were able to speak both from their own experience of sanitation and their perception of the experiences of their fellow community members. RAs used cognitive interviewing methods to determine if the enumerators understood the items as we intended them to be understood, asking them to explain, in their own words, what each item meant [53]. Modifications to the translations were made, as needed.

As an additional check to face validity, the RAs and the nine female enumerators piloted the items in a community similar to those where the data collection was to take place. During the pilot, the team noted items that were confusing to participants and wording that would improve understanding. The team met after the pilot to discuss feedback and amend the item translations one final time.

We selected 68 items for the final survey, 32 for urination and 36 for defecation. Items represented the three hypothesized domains: the physical environment (i.e., had difficulty finding a clean place to urinate); the social environment (i.e., worried people would talk about me if they saw me); and personal constraints that influence individual behavior (i.e., had difficulty or pain squatting for defecation). The survey asked women to indicate how often they had a particular experience within the previous 30 days, allowing them to provide one of four responses: never, sometimes, often or always.

### 2.4. Phase 2: Quantitative Research

The quantitative research phase involved three stages: a census, the creation of sampling frames and final sampling lists, and survey administration.

#### 2.4.1. Phase 2, Stage 1: Household Census

We administered a census to create sampling frames from which to identify participants eligible for the survey with the sanitation insecurity items (September–November 2014). We used a stratified, multistage, cluster sample design where we aimed to survey 1440 total participants from 60 communities (30 previously receiving an intervention and 30 previously serving as controls). Our sample size considered the application of our measure in later analyses, particularly to assess the impact of sanitation insecurity on various health outcomes. Therefore, the sample size was powered to detect small effect sizes using multilevel modeling (hierarchical modeling) across two levels: cluster level (i.e., intervention status), and individual level (i.e., latrine access, life stage, etc.) [54]. This sample size was based on a simulation study that demonstrated power to detect small (d = 0.20) direct and cross-level interaction effects for a continuous level-2 predictor to be greater than 96% for 60 clusters of 20 participants [55]. Hence, power was sufficient for both continuous and dichotomous predictors (base sample size of 1200). Our sample size of 1440 in 60 communities allowed for attrition due to incomplete surveys, error in census data leading to misidentification of eligible participants, and accidental double sampling of households.

Former intervention communities were eligible for inclusion in the survey, and therefore the census, if they had greater than 25% latrine coverage, and former control communities were eligible for inclusion if they had less than 20% latrine coverage. To select the eligible intervention communities, coverage data were used from the final trial data collection in December 2014, assuming little change in coverage over the course of nine months [43]. To select the eligible control communities, we sought feedback from a non-government organization partner actively working to provide sanitation in the control villages. Communities were excluded if they had been included in the qualitative activities that generated the survey items.

For the census, a team of trained enumerators asked a single representative from every household in each of the 60 communities to provide basic information about members of the household (sex, age, marital status) and the household itself (water and latrine access).

#### 2.4.2. Phase 2, Stage 2: Creation of Sampling Frames and Final Sampling Lists

We used data collected from the census to create sampling frames from which to select participants randomly for the final survey. As with the FLIs, we aimed to include women over 18 from four life stages: (1) unmarried; (2) married three years or less; (3) married over three years and age 49 or younger; and (4) women over 49 years of age of any marital status. From the census lists of each community, we used sex, age and marital status data for each individual community member to assign them to a life stage category. Individuals who did not belong to one of the four identified life stage categories were excluded (i.e., boys, men, girls under age 18). Four lists were generated per community, one for each life stage category. We randomly selected women to participate from each of these four lists.

#### 2.4.3. Phase 2, Stage 3: Survey Administration

Trained enumerators (those who assisted in the pilot phase of the items) administered the survey (December 2014 to February 2015, a year after the intervention ended). They collected data on sanitation insecurity items for the measure and on participant demographics, sanitation behavior and access, and mental health outcomes, which were evaluated in association with sanitation insecurity in a forthcoming paper.

In each community, the data collection team aimed to survey 24 women, six from each of the four life stage categories. Enumerators sought women in each life stage category list until the appropriate number of participants was attained, being mindful to not survey someone if another household member had already participated. Administration took approximately one hour. Data were collected on paper surveys and double entered.

### 2.5. Phase 3: Measurement Finalization

The measurement finalization phase followed the steps outlined in prior scale development studies [56] and involved three stages: exploratory factor analysis (EFA), confirmatory factor analysis (CFA), and assessment of differential item functioning (DIF). These three steps resulted in a final measure with a reduced number of items and identified sub-domains.

#### 2.5.1. Phase 3, Stage 1: Exploratory Factor Analysis

EFA is recommended as the first step of measure development when little or no research to determine the structure of a measure has been conducted [57]. After EFA is used to explore the factor structure of the data; CFA is recommended to test the factor structure identified in the EFA [57]. Because we had a large sample, we split our data into random sub-samples to first carry out EFA (*N*_1_ = 703) and then CFA (*N*_2_ = 705). We generated descriptive statistics of the demographic and household information provided by participants sampled and performed chi-square and *t*-tests to determine if there were any significant differences in demographic and household information between the sub-samples.

We estimated the frequencies of responses for all 68 sanitation insecurity items to determine distributions, both overall and across women in different life-stage groups. We also determined the skewness and kurtosis for each item. EFA does not require or assume that data be normal, however reporting of non-normality, minimal variation, and outliers is recommended [57].

With sub-sample *N*_1_ (703), we carried out EFA with all 68 items using MPLUS7 software (Muthén & Muthén, Los Angeles, CA, USA) to determine the number of factors and the factor loadings of each item [38]. We hypothesized that the factor structure would reflect the three broad domains previously identified: the physical environment, the social environment, and personal constraints. By default, MPLUS performs EFA by modeling all available data under the assumption of data missing at random [58]. As part of the EFA process, we estimated polychoric correlations of the items (i.e., correlations between observed ordinal variables) to assess the relationships between items [59,60]. We assumed the factors to be correlated and therefore selected an oblique rotation of the data (PROMAX) [57]. Due to the categorical responses, the estimator was WLSMV, a weighted least square parameter estimate that uses a diagonal weight matrix with standard errors and mean-and variance-adjusted chi-square test statistic [58]. The number of factors, factor loadings, and theoretical and model fit was assessed. We explored all factors with an eigen value greater than one (Kaiser Criteria) and decided a priori to drop any item with a factor loading <0.30 or if there were several cross-loaders (>0.50 on each factor) [61].

#### 2.5.2. Phase 3, Stage 2: Confirmatory Factor Analysis

After EFA, we used the second sub-sample (*N*_2_ = 705) to test the factor structure identified through EFA in MPLUS7 using the WLSMV estimator. Root mean square error of approximation (RMSEA), comparative fit index (CFI) and Tucker-Lewis index (TLI) were used to assess model fit. As with EFA, we dropped any item with a factor loading <0.30 or if it loaded on several factors (>0.05).

#### 2.5.3. Phase 3, Stage 1: Assessment of Differential Item Functioning (DIF)

After evaluating the CFA model, we assessed DIF to determine if women from the various life stage categories responded to individual items in the measure differently. Differential item functioning (DIF) occurs if sub-groups in the population have a different propensity to report specific responses, despite having the same underlying trait [62]. DIF can be problematic from a validity perspective; if sub-groups perform differently, inferences made from the measure may be biased [63]. In the present study, we were concerned that women at different life stages may respond differently because they may have interpretations or perspectives of an item that are specific to the life stage group to which they belong.

Using the same sub-sample used for the CFA (*N*_2_ = 705), we expanded the CFA model to Multiple Indicator Multiple Causes (MIMIC) structural equation models to test for DIF. First, we regressed the latent factors of the CFA model on three life stage covariates (with stage 1 (unmarried women)) serving as the reference category. No direct effects of life stage covariates on individual items were included. If there is a significant effect of covariates on latent factors, factor means are different for different covariate levels indicating population heterogeneity. Next, the output modification indices (MIs) (with MIs greater than 3.84), indicating significant improvement of model fit if modifications were to be made, were assessed to determine if allowing direct effects of any of the life stage covariates on individual items should be estimated freely. Direct effects of the covariates on specific items were added sequentially, starting with the direct effect associated with the largest modification index. After each addition, modification indices were re-assessed and additional direct effects were added until no DIF related MIs were generated. We assessed significance of each direct effect of the covariate on the respective item, which represents DIF. DIF can be addressed in two ways: account for DIF by generating individual scores through modeling approaches or eliminate items to create a DIF-free instrument [49]. We elected to eliminate items to make results comparable in future studies. CFA and MIMIC models were evaluated with MPLUS7 using a mean- and variance-adjusted weighted least squares (WLSMV) estimator as recommended for categorical data.

### 2.6. Sanitation Insecurity Factor Scores

We calculated final scores for each factor by calculating the sum of responses for each item (1 = never, 2 = sometimes, 3 = often, 4 = always) divided by the number of items in the factor (potential range for each factor: 1.0–4.0). Higher scores indicate a greater mean frequency of occurrence. We performed *t*-tests using STATA 14.0 (StataCorp LP, College Station, TX, USA) to determine if mean scores for each factor were significantly different by life stage and whether or not the woman had a functional household latrine.

### 2.7. Ethics

The Institutional Review Board at Emory University (Atlanta, GA, USA; IRB00072840) and the Institutional Ethics Committee of KIIT University (Bhubaneswar, India; KIMS/KIIT/IEC/795/2014) granted ethical approval for this study. After being informed of the details concerning the study, participants provided oral consent prior to participation, which was approved by both review committees.

## 3. Results

### 3.1. Participant Demographics

In total, 1437 surveys were administered. Twenty-nine women were eventually excluded because they were missing data for all relevant items (*N* = 1), had another household member already participate (*N* = 8), or were under age 18 (*N* = 20). The final sample size was 1408, including 341 (24%) unmarried women (stage 1), 320 (23%) women married three years or less (stage 2), 395 (28%) women married over three years (stage 3), and 352 (25%) women over age 49 of any marital status (stage 4).

Participants were 36 years old on average, almost all women were Hindu (99%), 48% reported to be general caste, 51% reported to be scheduled or “other backward” castes, less than 1% reported to be member of a scheduled tribe, and the majority had some schooling (76%) and a “below the poverty line” (BPL) card entitling them to government support (73%). Most participants had access to water outside their household compound (70%) and did not have household latrine access (58%) (Table 1).

### 3.2. Sanitation Insecurity Survey Items

The survey included 32 urination and 36 defecation items for potential inclusion in the final Sanitation Insecurity measure. The urination items to which participants most often responded “always” were those related to a lack of facility access, night, and concern for infection: “Felt concerned I would get an infection if I was urinating in an unsuitable/dirty place” (36%); “Felt concerned I would get an infection if I urinated on someone else’s urine” (34%); “Worried about not having a proper facility to urinate” (33%); “Felt scared urinating in the dark at night” (27%); and “Felt scared of ghosts when I went to urinate at night” (26%). The urination items to which participants most often responded “Never” were those related to experience of direct harms from others: 100% of respondents said they “never” “Had men or boys harm or harass me when going to urinate” and 99% of respondents said they “never” “Had people tease me when they saw me urinating” (Supplementary Table S1).

The defecation items to which participants most often responded “always” were those related to having and maintaining a toilet: “Worried about not having a toilet to defecate in” (54%) and “Worried that I have no money to build or maintain a toilet” (45%). The defecation items to which participants most commonly responded “never” were those related to experience of direct harms from others, like the urination items: 100% of respondents said they “never” “Had men or boys harm or harass me when going to defecate” and 99% of respondents said they “never” “Had people tease me when they saw me defecating” (99%) (See Supplementary Table S2). In an assessment of distributions, 12 urination items and 11 defecation items had skewness outside of the suggested ranges.

### 3.3. Exploratory Factor Analysis

Both sub-samples generated for the EFA and CFA analyses were similar overall and by life stage for all demographic information (no statistically significant differences were detected). During the EFA analysis, seven items showed little variance, resulting in negative correlations that prevented the EFA to run; these items, therefore, were eliminated and the EFA was then re-run.

We determined that the 7-factor solution with the PROMAX rotation best suited the data theoretically. The seven factors each produced strong and positive factor loadings and had good model fit (RMSEA = 0.035, should be <0.06 [44]; CFI and TLI results not provided for PROMAX rotation in MPLUS7) (Table 2). One item (“Changing and washing clothes used only for defecation increased workload”) was omitted because of multiple, low cross loadings and poor theoretical fit with the other factors, resulting in a total of 60 items among the seven factors.

The seven factors corresponded broadly to the three initially hypothesized domains: the physical environment, the social environment, and personal constraints that influence individual behavior. Specifically, factors 1, 4, and 7 largely concerned the physical environment. For factor 1, labeled “Potential harms”, all 11 items related to concerns or experiences about potential for harm at urination and defecation locations (i.e., risk of infection, polluting exposure to unclean places) (factor loadings: 0.697–0.910) (See Table 2 for all factor loadings). All four items in factor 4, “Night concerns” dealt with night, like fear of the dark or of ghosts (factor loadings: 0.722–0.870). The 12 items in factor 7 related to concerns about “defecation place”, including not having a toilet, needing to go far, dirty conditions, and lack of privacy (factor loadings: 0.683–0.945).

Factors 2 and 5 related to the social environment. Factor 2 was labeled “Social expectations and repercussions”. All 14 items in this factor dealt with a woman’s need to modify behaviors based on presence of others; suppression of urges based on social constraints; or concern about others talking about their behaviors if not socially acceptable (factor loadings: 0.533–0.863). Factor 5, “Social Support” included 6 items about women’s concerns providing or getting social support when they have a urination or defecation need, like finding support to look after work or dependents, or not being able to provide social support when addressing their needs (factor loadings: 0.481–0.933).

Factors 3 and 6 dealt with women’s personal constraints. Factor 3, labeled “Physical exertion or strain”, included nine items about concerns or experiences regarding how women needed to exert or strain their bodies to manage or control their urination and defecation needs, like withholding food and water to control urges, and doing work to wash the self or clothing after addressing needs (factor loadings: 0.431–0.715). Factor 6, “Physical Agility” included four items related to women’s personal physical agility when urinating or defecating, like difficulty squatting or concern for falling (factor loadings: 0.713–0.920).

### 3.4. Confirmatory Factor Analysis

For the CFA, two items were omitted, one due non-convergence for having a negative residual variance (“Had difficulty walking to defecation place”) and the second (“Had frequent pressure to urinate”) for having a very low factor loading (<0.150), resulting in a further refined model. Factor loadings for the 58 remaining items were significant and in similar ranges. All factors co–varied significantly and model fit was adequate.

### 3.5. Assessment of Differential Item Functioning

The final MIMIC model accommodated uniform DIF by allowing modifications to the model that allow life stage to have direct effects on specific items along with the indirect effects of the life stage covariates on the factor means. The final model included 10 suggested modifications involving the addition of direct effects on eight items (Supplementary Table S3). Despite these modifications, the indirect effects of life stage on the factor means did not change greatly. The most notable change was for “Physical exertion or strain” (Factor 3); women older than age 49 (stage 4) had a significantly lower factor mean (−0.140) than unmarried women (stage 1). All other previously reported significant differences by life stage remained the same with changes only made to the degree of difference.

The inclusion of the 10 suggested direct effects of life stages on specific items had little effect on model fit. Of the eight items that functioned differently, six pertained to women over 49 and two pertained to recently married women and women married over three years (See Supplementary Table S4).

### 3.6. Final Measure

We elected to delete the eight items that exhibited DIF to make the instrument more parsimonious and to allow general application across all life stages without having to adjust analysis for DIF. We did not feel that item deletion endangered construct validity given the range of items still remaining that address similar concepts. The final CFA model included 50 items (11 items in F1: “Potential harms”; 13 items in F2: “Social expectations and repercussions”; six items in F3: “Physical exertion or strain”; four items in F4: “Night Concerns”; four items in F5: “Social support”; six items in F6: “Physical agility”; and nine items in F7: “Defecation place”) (see Table 2 for final items by factor; See Appendix A for tool). All items loading on each factor were significant. The model fit was adequate, and slightly improved for CFI and TLI compared to the initial CFA (RMSEA = 0.060; CFI = 0.944; TLI = 0.941). All factors co-varied significantly (See Supplementary Table S3).

### 3.7. Sanitation Insecurity Scores

Mean sanitation insecurity scores for all women ranged from 1.11 (Factor 3: Physical exertion or strain) to 2.50 (Factor 4: Night concerns) (See Table 3). Scores were highest for unmarried women for five of the seven factors (Potential harms, Social expectations and repercussions, Physical exertion or strain, Night concerns, and Defecation Place). Scores were significantly higher than those of older women across all five of these factors, and were significantly higher than scores for married women and recently married women for four and three factors, respectively. Older women had significantly higher scores for the physical agility factor than unmarried women, indicating greater frequency of concerns for physical mobility associated with sanitation practices. Recently married women had significantly higher concerns about social support related to sanitation compared to unmarried women. Scores were significantly higher for six of the factors for women who did not have a functional household latrine.

## 4. Discussion

This study developed and validated a measure of sanitation insecurity to capture the existence and frequency of the full range of women’s concerns and negative experiences related to urination and defecation, which were previously unquantified. The rigorous, mixed methods approach utilized to produce this measure-including qualitative research, a census to identify appropriate respondents, and a survey involving over 1400 participants-was imperative to ensure that the final measurement items reflected and represented the voiced concerns and experiences of the target population. Further, the use of EFA to hypothesize the factor structure, CFA to evaluate it, and DIF to identify variability in response by life stage all served to strengthen the final measure. Results demonstrate that both life stage and ownership of a functional latrine can influence sanitation insecurity across all factors.

Bradley and Bartram outlined the need to re-think water security and include sanitation within their conceptual framing [64]. The authors note the need to consider provision (like access in other, previously noted definitions) and risk, including risks to sustainability and reliability, personal risks, risks related to place, and political, economic, and technical risks. Unlike the definitions previously described, this conceptualization does not highlight individual experiences of water and sanitation as influential to security, and how these experiences may be socially and culturally influenced. O’Reilly’s concept of *toilet security*, described previously, focuses primarily on availability and quality of facilities [37]. Like food and water insecurity research that has included experiences of insecurity, our research prioritizes individual perspectives and experiences. And as has been done with the experienced-based measures of water insecurity, our experienced–based measure of sanitation insecurity may be used to assess if and how the lived experiences of sanitation may lead to risks previously un–studied, by impacts mental health outcomes (see Wutich and Ragsdale [32] and Stevenson et al., [34]), despite facility access. A sanitation facility that is unbreakable, scalable, and technologically perfect is of no value if it is socially and culturally unacceptable, undignified, unsafe, inconvenient, and unfit for use. Recent research in Odisha has found that household and community level dynamics prevent women from making decisions about sanitation facilities for their households despite government and implementer efforts to do so [65]. Women continue to be left out of sanitation related discussions around facilities and their perspectives are under acknowledged [66,67]. Given the documented challenges women have faced with regard to their sanitation experiences and their gender-and sex-specific needs, a focus on understanding women’s experiences and whether or not sanitation technologies improve those experiences is imperative.

We proposed a measure of sanitation insecurity that reflected women’s voiced concerns about their sanitation experiences. The items reflected three broad domains: the physical environment, the social environment, and personal constraints. The seven factors that make up the final measure correspond to these three domains. It is imperative to note that only a few items actually correspond to sanitation technology (U01: worry about not having a proper facility to urinate and D01: worry about not having a toilet to defecate). Several items relate to concern about the physical environment (items in Factors 1, 4, and 7), however the construction of a toilet will not guarantee that these concerns are eliminated or even ameliorated unless engineers and practitioners make an intentional effort to address them. For example, concerns about harm from animals or people, fear at night, and the need to go a far distance (since facilities are typically outside the home) could be addressed by including women in decisions about the placement and design of facilities, but very well may not be. Further, as Routray notes, efforts to engage women should be evaluated to ensure that their voices are actually heard, especially since they may be in the room during sanitation–related activities but are not able to speak given cultural norms [65,68].

As with research on water and food insecurity, our sanitation insecurity measure included factors that specifically reflect the social needs women have that are related to sanitation and are not captured if focusing on assessment of facility availability alone. Issues related to the social environment (Factors 2 and 5) pose challenges for a WASH sector that historically has focused on engineering changes to the physical environment. From qualitative research, we know that women have difficulty addressing their urination and defecation needs if they have social constraints like work they are required to complete, restrictions on what time of day needs can be addressed, or depend on others to watch children [22,39,40]. Providing a toilet could ease these social difficulties if efforts to do so deliberately incorporate women’s needs and voiced concerns. However, from our research, mean scores for the social support factor were not significantly different for latrine owners compared to those who did not own a latrine, indicating that toilet access did not address the concerns in this domain. If a toilet is situated in a location considered by women to be accessible and contains the resources she needs within or attached to it (like water and a bathing area as appropriate), a woman may no longer need the assistance of others to care for dependents so she can take care of her own bodily (biological) needs. Involving women in decision making may help to ensure that facilities suit their needs, but these findings also suggest that sanitation programming should also extend beyond construction to impact and transform the gendered circumstances in which women are living. Referring back to Kandiyoti, women are operating within a set of constraints that influence their behaviors [38], including when they can attend to their needs and when they must attend to other responsibilities. Changing these constraints is no doubt challenging, but ignoring them likely will not result in improved circumstances for women if facilities alone remain the focus of the sanitation sector.

Personal constraints (Factors 3 and 5), namely those related to physical exertion, strain and agility, need further attention and could be addressed by mindful technology approaches. Mean scores for Factor 3, physical exertion or strain, were significantly different by life stage but not by ownership of a functional latrine, indicating limited capacity for latrines in this context to serve women’s needs. Two items in this factor ask women about controlling urination and defecation by withholding food and water. Women who undertake these practices are exerting agency over their circumstances in ways that are unhealthy yet give them some control of the constraints of their sanitation circumstances. For Factor 6, physical agility or strain, scores were significantly higher for older women and for those who did not own functional latrines. Women may need to exert tremendous amounts of energy to fetch water or clean themselves and their clothes post-defecation or may risk falling or experience pain squatting, particularly if they have limited mobility. These difficulties may be more pronounced if women suffer from urinary or fecal incontinence [69], have disabilities, are in advanced stages of pregnancy, or are elderly. In short, women may have different needs. Yet, in low-income settings where building facilities at scale is a priority, latrines are typically designed to accommodate the average, able–bodied user. Researchers have called for practitioners and policy makers to address the specific needs of users in the design of sanitation facilities for children at school (size of squatting holes, height of door knobs and locks, etc.), including school children with disabilities (ramps for wheelchair access) [70,71], and for women and girls who menstruate (water and space for washing and disposal units in stalls). While there are examples of initiatives that have prioritized children’s specific needs into sanitation designs (See, for example, the Design and construction manual for water supply and sanitary facilities in Ethiopian primary schools [72] among others), it remains unclear if these calls have had any substantial impact on the design of household or work facilities for women [73,74]. Similarly, practitioners and policy makers need to consider women’s personal abilities to use facilities, like their ability to walk or squat, when they are designed. Simple improvements, like the addition of an elevated commode or hand rails, would be easy and inexpensive to install. Government officials could promote these improvements and subsidize any additional costs that may be needed for those households that have members in need.

### Strengths and Limitations

This sanitation insecurity measure reflects the voiced concerns of women in various life stages in rural Odisha, India; it does not necessarily reflect those of other populations. As such, use of this tool in other settings may not be applicable; some of the items may not be relevant or there may be concerns and experiences in other populations that that are not captured. We recommend piloting or conducting cognitive interviews with the tool before broad use to identify irrelevant or missing items. Further research is needed to learn if this measure would be of use among other populations in India—whether with men and individuals younger than 18, or with women in urban and tribal areas—and beyond. Recent research in Odisha, India involving women in rural, urban, and tribal areas found that women in urban areas had higher sanitation related stressors that could be attributed to the environment or sexual coercion than women in other areas [22]. The evaluation of this tool with other populations could enable these populations to be compared and for intervention designs to target specific needs identified.

This tool does not capture seasonal variability, which may influence experiences. Data was collected in the winter months with only a 30-day re-call, and so it was not appropriate to ask women about concerns related to extreme heat or the monsoon, even though women described many concerns and challenges during these times. This tool should be used at different times of the year to see if the intensity of sanitation insecurity changes and additional questions could be considered for inclusion.

Due to its length, this tool poses challenges for practical use, particularly outside of the research setting. However, researchers and practitioners could use specific segments of the tool to answer questions about factors of interest. For example, if practitioners are particularly concerned about understanding and addressing the harms women perceive and experience when attending to their urination and defecation needs, only the items pertaining to Factor 1 (Potential Harms) could be administered.

This tool focuses on the negative experiences and perceptions that women have regarding urination and defecation; it does not assess the factors that may enable positive urination and defecation experiences. Further research that explores enabling factors is warranted. Finally, this tool does not capture menstruation-related concerns. A tool is under development to use in tandem as appropriate.

## 5. Conclusions

This sanitation insecurity measure aims to quantify the existence and frequency of the full range of women’s concerns and negative experiences related to sanitation. As with measures of food and water insecurity, this measure could be used to evaluate sanitation interventions to determine if they actually improve women’s experiences or if they have unintended consequences of making their experiences worse, therefore moving beyond simpler assessments that solely evaluate hardware and ability to contain feces. The measure can also be used to assess women’s experiences before a sanitation intervention is initiated in order to include components that actively address women’s sanitation concerns when the intervention is designed. This approach would allow those creating interventions to identify components that may not be addressed with hardware or technology components alone, but with messaging or activities that aim to change attitudes and norms to influence the social environments within which women are living. If a specific issue emerged as prevalent, for example suppression of urination or defecation to tend to household needs, practitioners could engage women on this issue, share that many other women have the same experience, and ask them to help identify strategies that may help remove barriers to tending to their needs. Finally, scores resulting from this measure could be used to determine if there is a relationship between women’s level of sanitation insecurity and their health, with attention to facets of health beyond infectious disease, like anxiety, quality of life and risk of violence.

## Figures and Tables

**Table 1:** Demographic characteristics of survey participants, overall and by life stage in Rural Orissa, India (N = 1408).

Characteristics	All		1. Unmarried (UM)		2. Recently Married (<3 Years) (RM)		3. Married (>3 Years) (M)		4. Over 49 (OW)	
Number of Participants	1408		341		320		395		352	
Village Status									
Control	707	50%	175	51%	162	51%	193	49%	177	50%
Intervention	701	50%	166	49%	158	49%	202	51%	175	50%
Age	36	(18)	21	(3)	24	(3)	35	(7)	63.6	(10)
Education									
None	335	24%	3	1%	7	2%	80	20%	245	70%
Some Primary	410	29%	53	16%	68	21%	190	48%	99	28%
Some Secondary	588	42%	235	69%	232	73%	114	29%	8	2%
Higher than Secondary	75	5%	50	15%	13	4%	12	3%	0	0%
Possession of Government Assistance Card ^1^										
Yes	1033	73%	259	76%	223	70%	286	73%	265	75%
Religion									
Hindu	1389	99%	339	99%	315	98%	386	98%	349	99%
Muslim	19	1%	2	1%	5	2%	9	2%	3	1%
Caste ^1^									
Brahmin	38	3%	10	3%	8	3%	12	3%	8	2%
Forward/General	672	48%	150	44%	151	47%	171	43%	155	44%
Scheduled Caste (SC)	247	18%	51	15%	59	19%	76	19%	61	17%
Other Backward Caste (OBC)	464	33%	124	37%	92	29%	128	32%	120	34%
Scheduled Tribe (ST)	11	1%	2	1%	2	1%	3	1%	4	1%
Don’t Know	19	1%	3	1%	7	2%	5	1%	4	1%
Has children	906	64%	0	1%	180	56%	382	97%	344	98%
Number of Children	2	(2)	0	(0)	1	(1)	2	(1)	5	(2)
Primary Drinking Water Source Location ^2^										
In Dwelling	131	10%	15	5%	43	15%	33	9%	40	12%
In Compound	273	20%	68	21%	71	24%	70	19%	64	19%
Outside Compound	927	70%	240	74%	183	62%	271	72%	233	69%
Household Latrine Ownership ^2^										
Yes	414	29%	71	21%	128	40%	103	26%	112	32%
No	815	58%	226	66%	155	48%	241	61%	193	55%
Under Construction	177	13%	44	13%	37	12%	49	13%	47	13%

Data are number and percent or mean and (standard deviation). ^1^ For *Possession of government Assistance Card*: Includes below Poverty Line (BPL) Card, Antodaya Card or both*. 1 missing* (*stage 3*); For *Caste*: 2 missing (stage 1 and stage 2) and 19 indicated don’t know; ^2^ For *Water source*: data taken from census, 77 participants with missing data; For *Latrine ownership*: data taken from census, 2 participants with missing data.

**Table 2:** Factor loadings for random split–half sample EFA (*N*_1_ = 703) and CFA models (*N*_2_ = 705), baseline and final MIMIC models (*N*_2_ = 708), and final CFA model (*N*_2_ = 708) with deletions based on DIF.

Factors and Associated Items	Item	Final EFA (*N*_1_ = 703)	CFA (*N*_2_ = 705)	Baseline MIMIC Model (*N*_2_ = 705)	Final MIMIC Model ^1^ (*N*_2_ = 705)	Final CFA ^2^ (*N*_2_ = 705)
**Factor 1:** *Potential Harms*						
Felt concerned I would get an infection if I was urinating in an unsuitable/dirty place	U10	0.822	0.963 *	0.962 *	0.962 *	0.964 *
Felt concerned I would get an infection if I urinated on someone elses urine	U15	0.810	0.953 *	0.951 *	0.951 *	0.956 *
Worried about getting an infection when going to defecate	D15	0.779	0.958 *	0.961 *	0.961 *	0.943 *
Had difficulty finding clean place to urinate	U06	0.773	0.883 *	0.889 *	0.889 *	0.885 *
Felt worried that I would step on urine	U08	0.758	0.853 *	0.856 *	0.856 *	0.855 *
Feared I would be harmed by someone when I went to urinate	U12	0.910	0.817 *	0.803 *	0.803 *	0.825 *
Worried about not having a proper facility to urinate	U01	0.697	0.825 *	0.830 *	0.830 *	0.824 *
Worried that someone would see me while urinating	U03	0.714	0.828 *	0.824 *	0.824 *	0.819 *
Feared I would be harmed by animals or insects when I went to urinate	U11	0.821	0.806 *	0.799 *	0.799 *	0.811 *
Feared I would be harmed by someone when I went to defecate	D26	0.706	0.798 *	0.808 *	0.808 *	0.794 *
Feared I would be harmed by animals or insects when I went to defecate	D12	0.791	0.724 *	0.733 *	0.733 *	0.717 *
**Factor 2:** *Social expectations resultant repercussions*						
Had difficulty finding a private place to urinate	U17	0.760	0.866 *	0.864 *	0.863 *	0.871 *
Had to suppress urge because people were around and could not go	U20	0.766	0.852 *	0.850 *	0.849 *	0.855 *
Worried people would talk about me if they saw me	D11	0.803	0.816 *	0.814 *	0.814 *	0.811 *
Worried people would talk about me if they saw me	U09	0.863	0.785 *	0.780 *	0.779 *	0.792 *
Had to suppress urge because I can only defecate at certain times of the day	D25	0.661	0.792 *	0.790 *	0.789 *	0.771 *
Had to stand while urinating because someone came	U25	0.610	0.760 *	0.762 *	0.761 *	0.752 *
Had trouble controlling urge to defecate	D28	0.553	0.700 *	0.729 *	0.728 *	0.695 *
Worried others would get upset if asked to accompany for urination	U31	0.856	0.707 *	0.704 *	0.703 *	0.684 *
Could not always go to urinate when there was a need	U02	0.766	0.680 *	0.695 *	0.694 *	0.681 *
Experienced difficulty controlling urge to urinate	U04	0.702	0.658 *	0.681 *	0.680 *	0.656 *
Had to suppress urge [to defecate] when workload was high	D09	0.563	0.656 *	0.655 *	0.654 *	0.636 *
Had to suppress when I got an urge at night	U32	0.704	0.593 *	0.598 *	0.597 *	0.586 *
Had to suppress [urination] when workload was high	U29	0.594	0.587 *	0.583 *	0.582 *	0.580 *
Had to suppress urge because did not have someone to accompany me ^✢^	U26	0.773	0.724 *	0.723 *	0.676 *	–
**Factor 3:** *Physical exertion or strain*						
Had difficulty accessing water for defecation	D21	0.674	0.906 *	0.882 *	0.884 *	0.915 *
Had difficulty accessing water for urination	U19	0.612	0.833 *	0.872 *	0.873 *	0.852 *
Had difficulty cleaning/washing myself after defecation	D23	0.709	0.835 *	0.825 *	0.828 *	0.848 *
Had to do extra work washing clothes because of dirty conditions where urinating	U23	0.715	0.758 *	0.755 *	0.758 *	0.752 *
Withheld food to control urge to defecate	D31	0.585	0.694 *	0.700 *	0.703 *	0.669 *
Withheld water to control urge to urinate	U28	0.387	0.567 *	0.568 *	0.571 *	0.564 *
Experienced pain during urination ^✢^	U05	0.601	0.583 *	0.639 *	0.636 *	–
Experienced pain during defecation ^✢^	D04	0.492	0.370 *	0.433 *	0.426 *	–
Had frequent pressure to urinate ^✢^	U22	0.431	–	–	–	–
**Factor 4:** *Night Concerns*						
Felt scared of ghosts when I went to urinate at night	U16	0.870	0.950 *	0.946 *	0.946 *	0.952 *
Felt scared urinating in the dark at night	U13	0.809	0.919 *	0.923 *	0.923 *	0.920 *
Felt scared defecating in the dark at night	D10	0.722	0.918 *	0.920 *	0.920 *	0.914 *
Felt scared of ghosts when I went to defecate at night	D18	0.793	0.915 *	0.918 *	0.918 *	0.914 *
**Factor 5:** *Social support*						
Had trouble finding someone to watch dependents (children, sick, elderly) so I could urinate	U27	0.928	0.939 *	0.945 *	0.956 *	0.962 *
Had trouble finding someone to watch dependents so I could defecate	D27	0.933	0.919 *	0.913 *	0.928 *	0.942 *
Worried about dependents (children, sick or elderly) who need me when I go to defecate	D33	0.920	0.906 *	0.905 *	0.918 *	0.933 *
Had to leave dependents (like children, sick, or elderly) alone to urinate	U24	0.907	0.889 *	0.880 *	0.897 *	0.915 *
Had to find someone to look after my work so I could defecate ^✢^	D20	0.619	0.791 *	0.769 *	0.881 *	–
Worried others would get upset if asked to accompany for defecation ^✢^	D32	0.481	0.876 *	0.867 *	1.033 *	–
**Factor 6:** *Physical agility*						
Had difficulty or pain squatting for defecation	D17	0.920	0.951 *	0.936 *	0.936 *	0.954 *
Had difficulty or pain sitting or getting up for urination	U18	0.878	0.925 *	0.920 *	0.920 *	0.920 *
Worried I would fall when going to defecate	D07	0.801	0.758 *	0.782 *	0.782 *	0.763 *
Had difficulty walking to defecation place ^✢^	D19	0.713	–	–	–	–
**Factor 7:** *Defecation place*						
Worried about defecating in the same place as others	D29	0.852	0.944 *	0.938 *	0.937 *	0.963 *
Worried about not having a toilet to defecate	D01	0.945	0.885 *	0.886 *	0.885 *	0.900 *
Worried have no money to build or maintain toilet	D34	0.913	0.872 *	0.875 *	0.874 *	0.890 *
Had difficulty finding a clean place to defecate	D05	0.879	0.876 *	0.884 *	0.884 *	0.888 *
Could not access preferred location	D06	0.739	0.865 *	0.870 *	0.869 *	0.885 *
Had to go back and forth to defecation location because could not find privacy	D36	0.765	0.860 *	0.874 *	0.873 *	0.860 *
Had to go far to defecate	D02	0.851	0.799 *	0.806 *	0.804 *	0.808 *
Defecation process/ activity of defecation took a long time to complete	D03	0.804	0.782 *	0.797 *	0.795 *	0.801 *
Had to do extra work washing clothes because of dirty conditions where defecating	D14	0.683	0.770 *	0.772 *	0.770 *	0.779 *
Worried that someone would see me defecating ^✢^	D08	0.828	0.869 *	0.859 *	0.813 *	–
Had to suppress the urge to defecate because people were around ^✢^	D24	0.799	0.851 *	0.845 *	0.821 *	–

^1^ Final MIMIC Model includes 10 modifications; ^2^ Includes deletions based on DIF; * *p* ≤ 0.050; ^✢^ Items in initial EFA model but removed during CFA because of a negative variance (D19) or low factor loading of <0.150 (U22), or later deleted as a result of DIF (U26, U05 D04, D20, D32, D08, D24).

**Table 3:** Sanitation Insecurity factor scores by life stage and ownership of a functional latrine.

Characteristics	Physical Environmental Factors			Social Environment Factors		Personal Constrains Factors	
	Factor 1: Potential Harms ^1^	Factor 4: Night Concerns	Factor 7: Defecation Place ^2^	Factor 2: Social Expectations & Repercussions ^3^	Factor 5: Social Support	Factor 3: Physical Exertion or Strain ^4^	Factor 6: Physical Agility
All	1.78 (0.76)	2.5 (1.09)	2.12 (0.91)	1.43 (0.43)	1.15 (0.43)	1.11 (0.28)	1.47 (0.75)
Life Stage						
Unmarried Women (Ref.)	1.99 (0.81)	2.56 (1.06)	2.28 (0.90)	1.52 (0.45)	1.04 (0.20)	1.14 (0.34)	1.20 (0.42)
Recently Married Women	1.86 (0.77) **	2.49 (1.12)	1.94 (0.97) **	1.49 (0.48)	1.41 (0.65) **	1.13 (0.33)	1.31 (0.58) *
Married Women	1.78 (0.74) **	2.07 (1.03) **	2.21 (0.92)	1.45 (0.41) *	1.16 (0.43) **	1.10 (0.24)	1.30 (0.55) *
Older Women	1.50 (0.64) **	1.67 (0.92) **	2.01 (0.83) **	1.27 (0.29) **	1.03 (0.15)	1.08 (0.18) *	2.01 (1.00) **
Ownership of Functional Latrine							
Owns (Ref.)	1.46 (0.61)	1.8 (1.00)	1.20 (0.41)	1.27 (0.34)	1.13 (0.40)	1.08 (0.22)	1.38 (0.72)
Does Not Own	1.95 (0.78) **	2.4 (1.08) **	2.62 (0.70) **	1.52 (0.44) **	1.17 (0.45)	1.14 (0.31) **	1.51 (0.77) *

Numbers are mean (SD); * *p* < 0.05; ** *p* < 0.001. ^1^ For F1, one participant had missing value and was removed (OW, No functional latrine); ^2^ For F7, one participant with missing values and was removed (MW, Functional latrine); ^3^ For F2, two participants had missing values and were removed (OW, Functional latrine; MW, No functional latrine); ^4^ For F3, one participant had missing values and was removed (OW, No functional latrine).

## Supplementary Materials

The following are available online at www.mdpi.com/1660-4601/14/7/755/s1, Table S1: Frequency of participant responses for urination module questions by random split halves and life stage categories; Table S2: Frequency of participant responses for defecation module questions by random split halves and life stage categories; Table S3: Factor loadings, factor co–variations, and model fit statistics for random split–half sample EFA (*N*_1_ = 703) and CFA models (*N*_2_ = 705), baseline and final MIMIC models (*N*_2_ = 708), and final CFA model (*N*_2_ = 708) with deletions based on DIF; Table S4: Structural Regressions, Direct Effects, and Model Fit Statistics of MIMIC Models (*N*_2_ = 705).

## Appendix A

The final Sanitation Insecurity tool is provided for adaptation and use below. For ease of use, items are organized not by factor, but by behavior.

**Table A1:** Sanitation Insecurity tool, presented with urination and defecation items grouped to facilitate delivery.

**Part A. Urination Module**						
**How Often Have You Experienced Any of the Following in the Previous 30 Days When Going to Urinate?**						
**F1**	**U01**	Worried about not having a proper facility to urinate	01 Never	02 Sometimes	03 Often	04 Always
**F2**	**U02**	Could not always go to urinate when there was a need	01	02	03	04
**F1**	**U03**	Worried that someone would see me while urinating	01	02	03	04
**F2**	**U04**	Experience difficulty controlling the urge to urinate	01	02	03	04
**F1**	**U05**	Had difficulty finding a clean place to urinate	01	02	03	04
**F1**	**U06**	Felt worried that I would step on urine	01	02	03	04
**F2**	**U07**	Worried people would talk about me if they saw me	01	02	03	04
**F1**	**U08**	Felt concerned I would get an infection if I was urinating in an unsuitable/dirty place	01	02	03	04
**F1**	**U09**	Feared I would be harmed by animals or insects when I went to urinate	01	02	03	04
**F1**	**U10**	Feared I would be harmed by someone when I went to urinate	01	02	03	04
**F4**	**U11**	Felt scared urinating in the dark at night	01	02	03	04
**F1**	**U12**	Felt concerned I would get an infection if I urinated on someone else's urine	01	02	03	04
**F4**	**U13**	Felt scared of ghosts when I went to urinate at night	01	02	03	04
**F2**	**U14**	Had difficulty finding a private place to urinate	01	02	03	04
**F6**	**U15**	Had difficulty or pain sitting or getting up for urination	01	02	03	04
**F3**	**U16**	Had difficulty accessing water for urination	01	02	03	04
**F2**	**U17**	Had to suppress urge because people were around and could not go	01	02	03	04
**F3**	**U18**	Had to do extra work washing clothes because of dirty conditions where urinating	01	02	03	04
**F5**	**U19**	Had to leave dependents (like children, sick or elderly) alone to urinate	01	02	03	04
**F2**	**U20**	Had to stand while urinating because someone came	01	02	03	04
**F5**	**U21**	Had trouble finding someone to watch dependents (like children, sick or elderly) so I could urinate	01	02	03	04
**F3**	**U22**	Withheld water to control urge to urinate	01	02	03	04
**F2**	**U23**	Had to suppress when workload was high	01	02	03	04
**F2**	**U24**	Worried others would get upset if asked to accompany for urination	01	02	03	04
**F2**	**U25**	Had to suppress when I got an urge at night	01	02	03	04
**Part B. Defecation Module**						
**How Often Have You Experienced Any of the Following in the Previous 30 Days When Going to Defecate?**						
**F7**	**D01**	Worried about not having toilet to defecate	01 Never	02 Sometimes	03 Often	04 Always
**F7**	**D02**	Had to go far to defecate	01	02	03	04
**F7**	**D03**	Defecation process/Activity of defecation took a long time to complete	01	02	03	04
**F7**	**D04**	Had difficulty finding a clean place to defecate	01	02	03	04
**F7**	**D05**	Could not access preferred location	01	02	03	04
**F6**	**D06**	Worried I would fall when going to defecate	01	02	03	04
**F2**	**D07**	Had to suppress urge when workload was high	01	02	03	04
**F4**	**D08**	Felt scared defecating in the dark at night	01	02	03	04
**F2**	**D09**	Worried people would talk about me if they saw me	01	02	03	04
**F1**	**D10**	Feared I would be harmed by animals or insects when I went to defecate	01	02	03	04
**F7**	**D11**	Had to do extra work washing clothes because of dirty conditions where defecating	01	02	03	04
**F1**	**D12**	Worried about getting an infection when going to defecate	01	02	03	04
**F6**	**D13**	Had difficulty or pain squatting for defecation	01	02	03	04
**F4**	**D14**	Felt scared of ghosts when I went to defecate at night	01	02	03	04
**F3**	**D15**	Had difficulty accessing water for defecation	01	02	03	04
**F3**	**D16**	Had difficulty cleaning/washing myself after defecation	01	02	03	04
**F2**	**D17**	Had to suppress urge because I can only defecate at certain times of the day	01	02	03	04
**F1**	**D18**	Feared I would be harmed by someone when I went to defecate	01	02	03	04
**F5**	**D19**	Had trouble finding someone to watch dependents (like children, sick or elderly) so I could defecate	01	02	03	04
**F2**	**D20**	Had trouble controlling urge to defecate	01	02	03	04
**F7**	**D21**	Worried about defecating in the same place as others	01	02	03	04
**F3**	**D22**	Withheld food to control urge to defecate	01	02	03	04
**F5**	**D23**	Worried about dependents (children, sick or elderly) who need me when I go to defecate	01	02	03	04
**F7**	**D24**	Worried that have no money to build or maintain toilet	01	02	03	04
**F7**	**D25**	Have had to go back and forth to defecation location because could not find privacy	01	02	03	04

Note: Both urination and defecation modules should be delivered collectively as factor items span both modules. Factors may be extracted for targeted use, but all relevant items across modules need to be delivered. Identifiers listed above (i.e., U01, U02, D01, etc. do not necessarily correspond to those noted in the paper. F1 = Potential harms Factor; F2 = Social expectations and repercussions Factor; F3 = Physical exertion or strain Factor; F4 = Night Concerns Factor; F5 = Social support Factor; F6 = Physical agility Factor; F7 = Defecation place Factor.
