# Supplementary material for: Assessing Women’s Negative Sanitation Experiences and Concerns: The Development of a Novel Sanitation Insecurity Measure
Source: Int J Environ Res Public Health. 2017 Jul 11;14(7):755. doi: 10.3390/ijerph14070755 (PMC5551193; doi:10.3390/ijerph14070755)
Supplement: Supplementary file 1 [file ijerph-14-00755-s001.docx]

| **Supplementary Table S1a: Frequency of participant responses for urination module questions by random split halves and life stage categories.**  ***Question:*** ***How often have you experienced any of the following in the previous 30 days when going to urinate?*** | | | | | | | | | | | | | | | | | | | | | | |
| --- | --- | --- | --- | --- | --- | --- | --- | --- | --- | --- | --- | --- | --- | --- | --- | --- | --- | --- | --- | --- | --- | --- |
|  | ***Full Sample*** | | ***Sub-Sample N_1_*** | | | | | | | | | | ***Sub-Sample N_2_*** | | | | | | | | | |
|  | All  N=1408 | | All  N_1_=703 | | 1. Unmarried (UM)  n=166 | | 2. Recently Married (RM) n=162 | | 3. Married (M)  n=185 | | 4. Over 49 (OW)  n=190 | | All  N_2_=705 | | 1. Unmarried (UM)  n=175 | | 2. Recently Married (RM) n=158 | | 3. Married (M)  n=210 | | 4. Over 49 (OW)  n=162 | |
|  |  |  |  |  |  |  |  |  |  |  |  |  |  |  |  |  |  |  |  |  |  |  |
| **u01 Worried about not having a proper facility to urinate^1^** | | | | | | | | | | | | | | | | | | | | | | |
| Never | 615 | 43.7% | 309 | 44.0% | 52 | 31.3% | 72 | 44.4% | 84 | 45.4% | 101 | 53.2% | 306 | 43.4% | 65 | 37.1% | 69 | 43.7% | 90 | 42.9% | 82 | 50.6% |
| Sometimes | 152 | 10.8% | 81 | 11.5% | 19 | 11.4% | 11 | 6.8% | 26 | 14.1% | 25 | 13.2% | 71 | 10.1% | 16 | 9.1% | 16 | 10.1% | 14 | 6.7% | 25 | 15.4% |
| Often | 184 | 13.1% | 99 | 14.1% | 24 | 14.5% | 22 | 13.6% | 27 | 14.6% | 26 | 13.7% | 85 | 12.1% | 22 | 12.6% | 14 | 8.9% | 29 | 13.8% | 20 | 12.3% |
| Always | 457 | 32.5% | 214 | 30.4% | 71 | 42.8% | 57 | 35.2% | 48 | 25.9% | 38 | 20.0% | 243 | 34.5% | 72 | 41.1% | 59 | 37.3% | 77 | 36.7% | 35 | 21.6% |
| **u02 Could not always go to urinate when there was a need^1^** | | | | | | | | | | | | | | | | | | | | | | |
| Never | 869 | 61.7% | 436 | 62.0% | 87 | 52.4% | 101 | 62.3% | 110 | 59.5% | 138 | 72.6% | 433 | 61.4% | 115 | 65.7% | 92 | 58.2% | 117 | 55.7% | 109 | 67.3% |
| Sometimes | 410 | 29.1% | 202 | 28.7% | 64 | 38.6% | 38 | 23.5% | 57 | 30.8% | 43 | 22.6% | 208 | 29.5% | 38 | 21.7% | 48 | 30.4% | 77 | 36.7% | 45 | 27.8% |
| Often | 98 | 7.0% | 44 | 6.3% | 11 | 6.6% | 12 | 7.4% | 13 | 7.0% | 8 | 4.2% | 54 | 7.7% | 20 | 11.4% | 15 | 9.5% | 12 | 5.7% | 7 | 4.3% |
| Always | 31 | 2.2% | 21 | 3.0% | 4 | 2.4% | 11 | 6.8% | 5 | 2.7% | 1 | 0.5% | 10 | 1.4% | 2 | 1.1% | 3 | 1.9% | 4 | 1.9% | 1 | 0.6% |
| **u03 Worried that someone would see me while urinating** | | | | | | | | | | | | | | | | | | | | | | |
| Never | 725 | 51.5% | 362 | 51.5% | 64 | 38.6% | 74 | 45.7% | 84 | 45.4% | 140 | 73.7% | 363 | 51.5% | 79 | 45.1% | 69 | 43.7% | 101 | 48.1% | 114 | 70.4% |
| Sometimes | 292 | 20.7% | 141 | 20.1% | 36 | 21.7% | 29 | 17.9% | 51 | 27.6% | 25 | 13.2% | 151 | 21.4% | 37 | 21.1% | 35 | 22.2% | 48 | 22.9% | 31 | 19.1% |
| Often | 163 | 11.6% | 82 | 11.7% | 31 | 18.7% | 21 | 13.0% | 24 | 13.0% | 6 | 3.2% | 81 | 11.5% | 16 | 9.1% | 24 | 15.2% | 33 | 15.7% | 8 | 4.9% |
| Always | 228 | 16.2% | 118 | 16.8% | 35 | 21.1% | 38 | 23.5% | 26 | 14.1% | 19 | 10.0% | 110 | 15.6% | 43 | 24.6% | 30 | 19.0% | 28 | 13.3% | 9 | 5.6% |
| **u04 Experience difficulty controlling urge to urinate** | | | | | | | | | | | | | | | | | | | | | | |
| Never | 982 | 69.7% | 488 | 69.4% | 104 | 62.7% | 104 | 64.2% | 128 | 69.2% | 152 | 80.0% | 494 | 69.7% | 123 | 70.3% | 102 | 64.6% | 151 | 71.9% | 118 | 72.8% |
| Sometimes | 318 | 22.6% | 164 | 23.3% | 49 | 29.5% | 43 | 26.5% | 43 | 23.2% | 29 | 15.3% | 154 | 21.7% | 37 | 21.1% | 40 | 25.3% | 41 | 19.5% | 36 | 22.2% |
| Often | 74 | 5.3% | 31 | 4.4% | 10 | 6.0% | 8 | 4.9% | 9 | 4.9% | 4 | 2.1% | 43 | 6.1% | 12 | 6.9% | 13 | 8.2% | 15 | 7.1% | 3 | 1.9% |
| Always | 34 | 2.4% | 20 | 2.8% | 3 | 1.8% | 7 | 4.3% | 5 | 2.7% | 5 | 2.6% | 14 | 2.0% | 3 | 1.7% | 3 | 1.9% | 3 | 1.4% | 5 | 3.1% |
| **u05 Experienced pain during urination** | | | | | | | | | | | | | | | | | | | | | | |
| Never | 1218 | 86.5% | 600 | 85.3% | 153 | 92.2% | 132 | 81.5% | 160 | 86.5% | 155 | 81.6% | 618 | 87.7% | 161 | 92.0% | 137 | 86.7% | 183 | 87.1% | 137 | 84.6% |
| Sometimes | 134 | 9.5% | 76 | 10.8% | 12 | 7.2% | 20 | 12.3% | 21 | 11.4% | 23 | 12.1% | 58 | 8.2% | 10 | 5.7% | 15 | 9.5% | 20 | 9.5% | 13 | 8.0% |
| Often | 25 | 1.8% | 11 | 1.6% | 0 | 0.0% | 7 | 4.3% | 0 | 0.0% | 4 | 2.1% | 14 | 2.0% | 2 | 1.1% | 3 | 1.9% | 3 | 1.4% | 6 | 3.7% |
| Always | 31 | 2.2% | 16 | 2.3% | 1 | 0.6% | 3 | 1.9% | 4 | 2.2% | 8 | 4.2% | 15 | 2.1% | 2 | 1.1% | 3 | 1.9% | 4 | 1.9% | 6 | 3.7% |
| **u06 Had difficulty finding a clean place to urinate** | | | | | | | | | | | | | | | | | | | | | | |
| Never | 774 | 55.0% | 396 | 56.3% | 73 | 44.0% | 92 | 56.8% | 104 | 56.2% | 127 | 66.8% | 378 | 53.6% | 85 | 48.6% | 82 | 51.9% | 110 | 52.4% | 101 | 62.3% |
| Sometimes | 225 | 16.0% | 113 | 16.1% | 32 | 19.3% | 22 | 13.6% | 31 | 16.8% | 28 | 14.7% | 112 | 15.9% | 26 | 14.9% | 25 | 15.8% | 32 | 15.2% | 29 | 17.6% |
| Often | 132 | 9.4% | 56 | 8.0% | 18 | 10.8% | 9 | 5.6% | 17 | 9.2% | 12 | 6.3% | 76 | 10.8% | 17 | 9.7% | 17 | 10.8% | 26 | 12.4% | 16 | 9.7% |
| Always | 277 | 19.7% | 138 | 19.6% | 43 | 25.9% | 39 | 24.1% | 33 | 17.8% | 23 | 12.1% | 139 | 19.7% | 47 | 26.9% | 34 | 21.5% | 42 | 20.0% | 16 | 9.7% |
| **u07 Felt afraid I would fall when going to urinate** | | | | | | | | | | | | | | | | | | | | | | |
| Never | 1246 | 88.5% | 626 | 89.0% | 159 | 95.8% | 154 | 95.1% | 176 | 95.1% | 137 | 72.1% | 620 | 87.9% | 166 | 94.9% | 144 | 91.1% | 195 | 92.9% | 115 | 71.0% |
| Sometimes | 93 | 6.6% | 44 | 6.3% | 4 | 2.4% | 5 | 3.1% | 9 | 4.9% | 26 | 13.7% | 49 | 7.0% | 8 | 4.6% | 11 | 7.0% | 9 | 4.3% | 21 | 13.0% |
| Often | 21 | 1.5% | 10 | 1.4% | 0 | 0.0% | 1 | 0.6% | 0 | 0.0% | 9 | 4.7% | 11 | 1.6% | 0 | 0.0% | 3 | 1.9% | 2 | 1.0% | 6 | 3.7% |
| Always | 48 | 3.4% | 23 | 3.3% | 3 | 1.8% | 2 | 1.2% | 0 | 0.0% | 18 | 9.5% | 25 | 3.5% | 1 | 0.6% | 0 | 0.0% | 4 | 1.9% | 20 | 12.3% |
| **u08 Felt worried that I would step on urine^2^** | | | | | | | | | | | | | | | | | | | | | | |
| Never | 873 | 62.0% | 448 | 63.8% | 82 | 49.4% | 98 | 60.5% | 118 | 63.8% | 150 | 79.4% | 425 | 60.3% | 93 | 53.1% | 87 | 55.1% | 127 | 60.5% | 118 | 72.8% |
| Sometimes | 255 | 18.1% | 125 | 17.8% | 38 | 22.9% | 30 | 18.5% | 35 | 18.9% | 22 | 11.6% | 130 | 18.4% | 36 | 20.6% | 29 | 18.4% | 38 | 18.1% | 27 | 16.7% |
| Often | 109 | 7.7% | 50 | 7.1% | 20 | 12.0% | 12 | 7.4% | 14 | 7.6% | 4 | 2.1% | 59 | 8.4% | 12 | 6.9% | 19 | 12.0% | 23 | 11.0% | 5 | 3.1% |
| Always | 170 | 12.1% | 79 | 11.3% | 26 | 15.7% | 22 | 13.6% | 18 | 9.7% | 13 | 6.9% | 91 | 12.9% | 34 | 19.4% | 23 | 14.6% | 22 | 10.5% | 12 | 7.4% |
| **u09 Worried people would talk about me if they saw me** | | | | | | | | | | | | | | | | | | | | | | |
| Never | 1115 | 79.2% | 561 | 79.8% | 112 | 67.5% | 118 | 72.8% | 154 | 83.2% | 177 | 93.2% | 554 | 78.6% | 129 | 73.7% | 121 | 76.6% | 162 | 77.1% | 142 | 87.7% |
| Sometimes | 103 | 7.3% | 47 | 6.7% | 15 | 9.0% | 17 | 10.5% | 9 | 4.9% | 6 | 3.2% | 56 | 7.9% | 16 | 9.1% | 12 | 7.6% | 14 | 6.7% | 14 | 8.6% |
| Often | 83 | 5.9% | 38 | 5.4% | 17 | 10.2% | 7 | 4.3% | 10 | 5.4% | 4 | 2.1% | 45 | 6.4% | 12 | 6.9% | 8 | 5.1% | 21 | 10.0% | 4 | 2.5% |
| Always | 107 | 7.6% | 57 | 8.1% | 22 | 13.3% | 20 | 12.3% | 12 | 6.5% | 3 | 1.6% | 50 | 7.1% | 18 | 10.3% | 17 | 10.8% | 13 | 6.2% | 2 | 1.2% |
| **Supplementary Table S1b: Frequency of participant responses for urination module questions by random split halves and life stage categories. *(Continued).***  ***Question:*** ***How often have you experienced any of the following in the previous 30 days when going to urinate?*** | | | | | | | | | | | | | | | | | | | | | | |
|  | ***Full Sample*** | | ***Sub-Sample N_1_*** | | | | | | | | | | ***Sub-Sample N_2_*** | | | | | | | | | |
|  | All  N=1408 | | All  N_1_=703 | | 1. Unmarried (UM)  n=166 | | 2. Recently Married (RM) n=162 | | 3. Married (M)  n=185 | | 4. Over 49 (OW)  n=190 | | All  N_2_=705 | | 1. Unmarried (UM)  n=175 | | 2. Recently Married (RM) n=158 | | 3. Married (M)  n=210 | | 4. Over 49 (OW)  n=162 | |
|  |  |  |  |  |  |  |  |  |  |  |  |  |  |  |  |  |  |  |  |  |  |  |
| **u10 Felt concerned I would get an infection if I was urinating in an unsuitable/ dirty place^2^** | | | | | | | | | | | | | | | | | | | | | | |
| Never | 581 | 41.3% | 296 | 42.1% | 51 | 30.7% | 54 | 33.3% | 74 | 40.0% | 117 | 62% | 285 | 40.4% | 54 | 30.9% | 54 | 34.2% | 82 | 39.0% | 95 | 58.6% |
| Sometimes | 190 | 13.5% | 102 | 14.5% | 23 | 13.9% | 20 | 12.3% | 33 | 17.8% | 26 | 14% | 88 | 12.5% | 21 | 12.0% | 20 | 12.7% | 26 | 12.4% | 21 | 13.0% |
| Often | 132 | 9.4% | 78 | 11.1% | 22 | 13.3% | 20 | 12.3% | 24 | 13.0% | 11 | 6% | 55 | 7.8% | 16 | 9.1% | 7 | 4.4% | 21 | 10.0% | 11 | 6.8% |
| Always | 505 | 35.9% | 228 | 32.4% | 70 | 42.2% | 68 | 42.0% | 54 | 29.2% | 36 | 19% | 277 | 39.3% | 84 | 48.0% | 77 | 48.7% | 81 | 38.6% | 35 | 21.6% |
| **u11 Feared I would be harmed by animals or insects when I went to urinate** | | | | | | | | | | | | | | | | | | | | | | |
| Never | 1166 | 82.8% | 580 | 82.5% | 133 | 80.1% | 134 | 82.7% | 152 | 82.2% | 161 | 83.9% | 586 | 83.1% | 134 | 76.1% | 130 | 82.3% | 173 | 82.4% | 149 | 92.0% |
| Sometimes | 123 | 8.7% | 60 | 8.5% | 16 | 9.6% | 17 | 10.5% | 15 | 8.1% | 12 | 6.3% | 63 | 8.9% | 18 | 10.2% | 17 | 10.8% | 19 | 9.0% | 9 | 5.6% |
| Often | 18 | 1.3% | 11 | 1.6% | 4 | 2.4% | 1 | 0.6% | 5 | 2.7% | 1 | 0.5% | 7 | 1.0% | 3 | 1.7% | 2 | 1.3% | 2 | 1.0% | 0 | 0.0% |
| Always | 101 | 7.2% | 52 | 7.4% | 13 | 7.8% | 10 | 6.2% | 13 | 7.0% | 16 | 8.3% | 49 | 7.0% | 20 | 11.4% | 9 | 5.7% | 16 | 7.6% | 4 | 2.5% |
| **u12 Feared I would be harmed by someone when I went to urinate** | | | | | | | | | | | | | | | | | | | | | | |
| Never | 1294 | 91.9% | 651 | 92.6% | 153 | 92.2% | 152 | 93.8% | 170 | 91.9% | 176 | 92.6% | 643 | 91.2% | 149 | 85.1% | 145 | 91.8% | 192 | 91.4% | 157 | 96.9% |
| Sometimes | 48 | 3.4% | 20 | 2.8% | 4 | 2.4% | 5 | 3.1% | 7 | 3.8% | 4 | 2.1% | 28 | 4.0% | 8 | 4.6% | 5 | 3.2% | 12 | 5.7% | 3 | 1.9% |
| Often | 8 | 0.6% | 4 | 0.6% | 2 | 1.2% | 0 | 0.0% | 2 | 1.1% | 0 | 0.0% | 4 | 0.6% | 2 | 1.1% | 1 | 0.6% | 1 | 0.5% | 0 | 0.0% |
| Always | 58 | 4.1% | 28 | 4.0% | 7 | 4.2% | 5 | 3.1% | 6 | 3.2% | 10 | 5.3% | 30 | 4.3% | 16 | 9.1% | 7 | 4.4% | 5 | 2.4% | 2 | 1.2% |
| **u13 Felt scared urinating in the dark at night** | | | | | | | | | | | | | | | | | | | | | | |
| Never | 628 | 44.6% | 322 | 45.8% | 54 | 32.5% | 61 | 37.7% | 86 | 46.5% | 121 | 63.7% | 306 | 43.4% | 49 | 28.0% | 50 | 31.6% | 101 | 48.1% | 106 | 65.4% |
| Sometimes | 246 | 17.5% | 126 | 17.9% | 32 | 19.3% | 24 | 14.8% | 40 | 21.6% | 30 | 15.8% | 120 | 17.0% | 30 | 17.1% | 20 | 12.7% | 43 | 20.5% | 27 | 16.7% |
| Often | 156 | 11.1% | 74 | 10.5% | 23 | 13.9% | 25 | 15.4% | 21 | 11.4% | 5 | 2.6% | 82 | 11.6% | 23 | 13.1% | 27 | 17.1% | 26 | 12.4% | 6 | 3.7% |
| Always | 378 | 26.8% | 181 | 25.7% | 57 | 34.3% | 52 | 32.1% | 38 | 20.5% | 34 | 17.9% | 197 | 27.9% | 73 | 41.7% | 61 | 38.6% | 40 | 19.0% | 23 | 14.2% |
| **u14 Had people tease me when they saw me urinating** | | | | | | | | | | | | | | | | | | | | | | |
| Never | 1393 | 98.9% | 697 | 99.1% | 164 | 98.8% | 161 | 99.4% | 183 | 98.9% | 189 | 99.5% | 696 | 98.7% | 172 | 98.3% | 153 | 96.8% | 209 | 99.5% | 162 | 100% |
| Sometimes | 10 | 0.7% | 4 | 0.6% | 1 | 0.6% | 1 | 0.6% | 1 | 0.5% | 1 | 0.5% | 6 | 0.9% | 2 | 1.1% | 3 | 1.9% | 1 | 0.5% | 0 | 0.0% |
| Often | 3 | 0.2% | 2 | 0.3% | 1 | 0.6% | 0 | 0.0% | 1 | 0.5% | 0 | 0.0% | 1 | 0.1% | 0 | 0.0% | 1 | 0.6% | 0 | 0.0% | 0 | 0.0% |
| Always | 2 | 0.1% | 0 | 0.0% | 0 | 0.0% | 0 | 0.0% | 0 | 0.0% | 0 | 0.0% | 2 | 0.3% | 1 | 0.6% | 1 | 0.6% | 0 | 0.0% | 0 | 0.0% |
| **u15 Felt concerned I would get an infection if urinated on someone else's urine** | | | | | | | | | | | | | | | | | | | | | | |
| Never | 581 | 41.3% | 300 | 42.7% | 52 | 31.3% | 54 | 33.3% | 75 | 40.5% | 119 | 62.6% | 281 | 39.9% | 54 | 30.9% | 54 | 34.2% | 82 | 39.0% | 91 | 56.2% |
| Sometimes | 200 | 14.2% | 105 | 14.9% | 22 | 13.3% | 22 | 13.6% | 37 | 20.0% | 24 | 12.6% | 95 | 13.5% | 19 | 10.9% | 18 | 11.4% | 33 | 15.7% | 25 | 15.4% |
| Often | 145 | 10.3% | 76 | 10.8% | 22 | 13.3% | 16 | 9.9% | 23 | 12.4% | 15 | 7.9% | 69 | 9.8% | 21 | 12.0% | 12 | 7.6% | 19 | 9.0% | 17 | 10.5% |
| Always | 482 | 34.2% | 222 | 31.6% | 70 | 42.2% | 70 | 43.2% | 50 | 27.0% | 32 | 16.8% | 260 | 36.9% | 81 | 46.3% | 74 | 46.8% | 76 | 36.2% | 29 | 17.9% |
| **u16 Felt scared of ghosts when I went to urinate at night** | | | | | | | | | | | | | | | | | | | | | | |
| Never | 648 | 46.0% | 326 | 46.4% | 47 | 28.3% | 58 | 35.8% | 92 | 49.7% | 129 | 67.9% | 322 | 45.7% | 53 | 30.3% | 50 | 31.6% | 101 | 48.1% | 118 | 72.8% |
| Sometimes | 246 | 17.5% | 130 | 18.5% | 35 | 21.1% | 27 | 16.7% | 37 | 20.0% | 31 | 16.3% | 116 | 16.5% | 33 | 18.9% | 27 | 17.1% | 37 | 17.6% | 19 | 11.7% |
| Often | 149 | 10.6% | 69 | 9.8% | 30 | 18.1% | 16 | 9.9% | 17 | 9.2% | 6 | 3.2% | 80 | 11.3% | 23 | 13.1% | 21 | 13.3% | 27 | 12.9% | 9 | 5.6% |
| Always | 365 | 25.9% | 178 | 25.3% | 54 | 32.5% | 61 | 37.7% | 39 | 21.1% | 24 | 12.6% | 187 | 26.5% | 66 | 37.7% | 60 | 38.0% | 45 | 21.4% | 16 | 9.9% |
| **u17 Had difficulty finding a private place to urinate** | | | | | | | | | | | | | | | | | | | | | | |
| Never | 736 | 52.3% | 373 | 53.1% | 68 | 41.0% | 83 | 51.2% | 97 | 52.4% | 125 | 65.8% | 363 | 51.5% | 80 | 45.7% | 78 | 49.4% | 107 | 51.0% | 98 | 59.8% |
| Sometimes | 316 | 22.4% | 150 | 21.3% | 37 | 22.3% | 27 | 16.7% | 47 | 25.4% | 39 | 20.5% | 166 | 23.5% | 39 | 22.3% | 35 | 22.2% | 46 | 21.9% | 46 | 28.0% |
| Often | 190 | 13.5% | 91 | 12.9% | 31 | 18.7% | 19 | 11.7% | 26 | 14.1% | 15 | 7.9% | 99 | 14.0% | 31 | 17.7% | 23 | 14.6% | 34 | 16.2% | 11 | 6.7% |
| Always | 166 | 11.8% | 89 | 12.7% | 30 | 18.1% | 33 | 20.4% | 15 | 8.1% | 11 | 5.8% | 77 | 10.9% | 25 | 14.3% | 22 | 13.9% | 23 | 11.0% | 7 | 4.3% |
| **u18 Had difficulty or pain sitting or getting up for urination** | | | | | | | | | | | | | | | | | | | | | | |
| Never | 981 | 69.7% | 488 | 69.4% | 132 | 79.5% | 121 | 74.7% | 151 | 81.6% | 84 | 44.2% | 493 | 69.9% | 150 | 85.7% | 120 | 75.9% | 150 | 71.4% | 73 | 45.1% |
| Sometimes | 229 | 16.3% | 108 | 15.4% | 27 | 16.3% | 22 | 13.6% | 20 | 10.8% | 39 | 20.5% | 121 | 17.2% | 21 | 12.0% | 24 | 15.2% | 44 | 21.0% | 32 | 19.8% |
| Often | 75 | 5.3% | 39 | 5.5% | 3 | 1.8% | 10 | 6.2% | 8 | 4.3% | 18 | 9.5% | 36 | 5.1% | 1 | 0.6% | 6 | 3.8% | 7 | 3.3% | 22 | 13.6% |
| Always | 123 | 8.7% | 68 | 9.7% | 4 | 2.4% | 9 | 5.6% | 6 | 3.2% | 49 | 25.8% | 55 | 7.8% | 3 | 1.7% | 8 | 5.1% | 9 | 4.3% | 35 | 21.6% |
| **Supplementary Table S1c: Frequency of participant responses for urination module questions by random split halves and life stage categories. *(Continued).***  ***Question:*** ***How often have you experienced any of the following in the previous 30 days when going to urinate?*** | | | | | | | | | | | | | | | | | | | | | | |
|  | ***Full Sample*** | | ***Sub-Sample N_1_*** | | | | | | | | | | ***Sub-Sample N_2_*** | | | | | | | | | |
|  | All  N=1408 | | All  N_1_=703 | | 1. Unmarried (UM)  n=166 | | 2. Recently Married (RM) n=162 | | 3. Married (M)  n=185 | | 4. Over 49 (OW)  n=190 | | All  N_2_=705 | | 1. Unmarried (UM)  n=175 | | 2. Recently Married (RM) n=158 | | 3. Married (M)  n=210 | | 4. Over 49 (OW)  n=162 | |
|  |  |  |  |  |  |  |  |  |  |  |  |  |  |  |  |  |  |  |  |  |  |  |
| **u19 Had difficulty accessing water for urination** | | | | | | | | | | | | | | | | | | | | | | |
| Never | 1353 | 96.1% | 677 | 96.3% | 158 | 95.2% | 158 | 97.5% | 177 | 95.7% | 184 | 96.8% | 676 | 95.9% | 169 | 96.6% | 153 | 96.8% | 201 | 95.7% | 153 | 94.4% |
| Sometimes | 37 | 2.6% | 18 | 2.6% | 5 | 3.0% | 4 | 2.5% | 5 | 2.7% | 4 | 2.1% | 19 | 2.7% | 3 | 1.7% | 2 | 1.3% | 6 | 2.9% | 8 | 4.9% |
| Often | 5 | 0.4% | 2 | 0.3% | 1 | 0.6% | 0 | 0.0% | 0 | 0.0% | 1 | 0.5% | 3 | 0.4% | 1 | 0.6% | 0 | 0.0% | 1 | 0.5% | 1 | 0.6% |
| Always | 13 | 0.9% | 6 | 0.9% | 2 | 1.2% | 0 | 0.0% | 3 | 1.6% | 1 | 0.5% | 7 | 1.0% | 2 | 1.1% | 3 | 1.9% | 2 | 1.0% | 0 | 0.0% |
| **u20 Had to suppress urge because people were around and could not go** | | | | | | | | | | | | | | | | | | | | | | |
| Never | 703 | 49.9% | 359 | 51.1% | 62 | 37.3% | 79 | 48.8% | 90 | 48.6% | 128 | 67.4% | 344 | 48.8% | 70 | 40.0% | 81 | 51.3% | 95 | 45.2% | 98 | 60.5% |
| Sometimes | 581 | 41.3% | 284 | 40.4% | 83 | 50.0% | 64 | 39.5% | 79 | 42.7% | 58 | 30.5% | 297 | 42.1% | 84 | 48.0% | 59 | 37.3% | 94 | 44.8% | 60 | 37.0% |
| Often | 102 | 7.2% | 50 | 7.1% | 16 | 9.6% | 17 | 10.5% | 14 | 7.6% | 3 | 1.6% | 52 | 7.4% | 18 | 10.3% | 16 | 10.1% | 14 | 6.7% | 4 | 2.5% |
| Always | 22 | 1.6% | 10 | 1.4% | 5 | 3.0% | 2 | 1.2% | 2 | 1.1% | 1 | 0.5% | 12 | 1.7% | 3 | 1.7% | 2 | 1.3% | 7 | 3.3% | 0 | 0.0% |
| **u21 Had difficulty walking to urination place** | | | | | | | | | | | | | | | | | | | | | | |
| Never | 1317 | 93.5% | 660 | 93.9% | 159 | 95.8% | 155 | 95.7% | 177 | 95.7% | 169 | 88.9% | 657 | 93.2% | 169 | 96.6% | 152 | 96.2% | 197 | 93.8% | 139 | 85.8% |
| Sometimes | 54 | 3.8% | 25 | 3.6% | 3 | 1.8% | 5 | 3.1% | 6 | 3.2% | 11 | 5.8% | 29 | 4.1% | 4 | 2.3% | 5 | 3.2% | 10 | 4.8% | 10 | 6.2% |
| Often | 14 | 1.0% | 5 | 0.7% | 1 | 0.6% | 1 | 0.6% | 0 | 0.0% | 3 | 1.6% | 9 | 1.3% | 1 | 0.6% | 1 | 0.6% | 1 | 0.5% | 6 | 3.7% |
| Always | 23 | 1.6% | 13 | 1.8% | 3 | 1.8% | 1 | 0.6% | 2 | 1.1% | 7 | 3.7% | 10 | 1.4% | 1 | 0.6% | 0 | 0.0% | 2 | 1.0% | 7 | 4.3% |
| **u22 Had frequent pressure to urinate** | | | | | | | | | | | | | | | | | | | | | | |
| Never | 1036 | 73.6% | 518 | 73.7% | 141 | 84.9% | 123 | 75.9% | 130 | 70.3% | 124 | 65.3% | 518 | 73.5% | 148 | 84.6% | 112 | 70.9% | 156 | 74.3% | 102 | 63.0% |
| Sometimes | 140 | 9.9% | 69 | 9.8% | 11 | 6.6% | 14 | 8.6% | 26 | 14.1% | 18 | 9.5% | 71 | 10.1% | 11 | 6.3% | 20 | 12.7% | 21 | 10.0% | 19 | 11.7% |
| Often | 98 | 7.0% | 44 | 6.3% | 7 | 4.2% | 9 | 5.6% | 14 | 7.6% | 14 | 7.4% | 54 | 7.7% | 7 | 4.0% | 9 | 5.7% | 20 | 9.5% | 18 | 11.1% |
| Always | 134 | 9.5% | 72 | 10.2% | 7 | 4.2% | 16 | 9.9% | 15 | 8.1% | 34 | 17.9% | 62 | 8.8% | 9 | 5.1% | 17 | 10.8% | 13 | 6.2% | 23 | 14.2% |
| **u23 Had to do extra work washing clothes because of dirty conditions where urinating** | | | | | | | | | | | | | | | | | | | | | | |
| Never | 1331 | 94.5% | 665 | 94.6% | 154 | 92.8% | 155 | 95.7% | 171 | 92.4% | 185 | 97.4% | 666 | 94.5% | 162 | 92.6% | 151 | 95.6% | 198 | 94.3% | 155 | 95.7% |
| Sometimes | 62 | 4.4% | 30 | 4.3% | 10 | 6.0% | 6 | 3.7% | 11 | 5.9% | 3 | 1.6% | 32 | 4.5% | 12 | 6.9% | 3 | 1.9% | 10 | 4.8% | 7 | 4.3% |
| Often | 4 | 0.3% | 1 | 0.1% | 0 | 0.0% | 0 | 0.0% | 1 | 0.5% | 0 | 0.0% | 3 | 0.4% | 0 | 0.0% | 2 | 1.3% | 1 | 0.5% | 0 | 0.0% |
| Always | 11 | 0.8% | 7 | 1.0% | 2 | 1.2% | 1 | 0.6% | 2 | 1.1% | 2 | 1.1% | 4 | 0.6% | 1 | 0.6% | 2 | 1.3% | 1 | 0.5% | 0 | 0.0% |
| **u24 Had to leave dependents (like children, sick or elderly) alone to urinate** | | | | | | | | | | | | | | | | | | | | | | |
| Never | 1242 | 88.2% | 631 | 89.8% | 160 | 96.4% | 116 | 71.6% | 168 | 90.8% | 187 | 98.4% | 611 | 86.7% | 167 | 95.4% | 110 | 69.6% | 177 | 84.3% | 157 | 96.9% |
| Sometimes | 114 | 8.1% | 52 | 7.4% | 6 | 3.6% | 29 | 17.9% | 14 | 7.6% | 3 | 1.6% | 62 | 8.8% | 5 | 2.9% | 30 | 19.0% | 23 | 11.0% | 4 | 2.5% |
| Often | 41 | 2.9% | 17 | 2.4% | 0 | 0.0% | 14 | 8.6% | 3 | 1.6% | 0 | 0.0% | 24 | 3.4% | 0 | 0.0% | 14 | 8.9% | 9 | 4.3% | 1 | 0.6% |
| Always | 11 | 0.8% | 3 | 0.4% | 0 | 0.0% | 3 | 1.9% | 0 | 0.0% | 0 | 0.0% | 8 | 1.1% | 3 | 1.7% | 4 | 2.5% | 1 | 0.5% | 0 | 0.0% |
| **u25 Had to stand while urinating because someone came** | | | | | | | | | | | | | | | | | | | | | | |
| Never | 671 | 47.7% | 346 | 49.2% | 59 | 35.5% | 91 | 56.2% | 64 | 34.6% | 132 | 69.5% | 325 | 46.1% | 75 | 42.9% | 67 | 42.4% | 82 | 39.0% | 101 | 62.3% |
| Sometimes | 659 | 46.8% | 320 | 45.5% | 95 | 57.2% | 62 | 38.3% | 108 | 58.4% | 55 | 28.9% | 339 | 48.1% | 92 | 52.6% | 75 | 47.5% | 118 | 56.2% | 54 | 33.3% |
| Often | 71 | 5.0% | 34 | 4.8% | 10 | 6.0% | 9 | 5.6% | 12 | 6.5% | 3 | 1.6% | 37 | 5.2% | 7 | 4.0% | 15 | 9.5% | 8 | 3.8% | 7 | 4.3% |
| Always | 7 | 0.5% | 3 | 0.4% | 2 | 1.2% | 0 | 0.0% | 1 | 0.5% | 0 | 0.0% | 4 | 0.6% | 1 | 0.6% | 1 | 0.6% | 2 | 1.0% | 0 | 0.0% |
| **u26 Had to suppress urge because did not have someone to accompany me^2^** | | | | | | | | | | | | | | | | | | | | | | |
| Never | 1177 | 83.7% | 590 | 84.0% | 115 | 70.1% | 130 | 80.2% | 163 | 88.1% | 182 | 95.8% | 587 | 83.3% | 133 | 76.0% | 119 | 75.3% | 181 | 86.2% | 154 | 95.1% |
| Sometimes | 199 | 14.1% | 97 | 13.8% | 44 | 26.8% | 26 | 16.0% | 19 | 10.3% | 8 | 4.2% | 102 | 14.5% | 33 | 18.9% | 33 | 20.9% | 28 | 13.3% | 8 | 4.9% |
| Often | 25 | 1.8% | 14 | 2.0% | 5 | 3.0% | 6 | 3.7% | 3 | 1.6% | 0 | 0.0% | 11 | 1.6% | 7 | 4.0% | 3 | 1.9% | 1 | 0.5% | 0 | 0.0% |
| Always | 6 | 0.4% | 1 | 0.1% | 1 | 0.6% | 0 | 0.0% | 0 | 0.0% | 0 | 0.0% | 5 | 0.7% | 2 | 1.1% | 3 | 1.9% | 0 | 0.0% | 0 | 0.0% |
| **u27 Had trouble finding someone to watch dependents (like children, sick or elderly) so I could urinate** | | | | | | | | | | | | | | | | | | | | | | |
| Never | 1287 | 91.4% | 651 | 92.6% | 161 | 97.0% | 128 | 79.0% | 173 | 93.5% | 189 | 99.5% | 636 | 90.2% | 174 | 99.4% | 121 | 76.6% | 182 | 86.7% | 159 | 98.1% |
| Sometimes | 95 | 6.7% | 44 | 6.3% | 5 | 3.0% | 27 | 16.7% | 11 | 5.9% | 1 | 0.5% | 51 | 7.2% | 1 | 0.6% | 26 | 16.5% | 22 | 10.5% | 2 | 1.2% |
| Often | 20 | 1.4% | 5 | 0.7% | 0 | 0.0% | 5 | 3.1% | 0 | 0.0% | 0 | 0.0% | 15 | 2.1% | 0 | 0.0% | 9 | 5.7% | 5 | 2.4% | 1 | 0.6% |
| Always | 6 | 0.4% | 3 | 0.4% | 0 | 0.0% | 2 | 1.2% | 1 | 0.5% | 0 | 0.0% | 3 | 0.4% | 0 | 0.0% | 2 | 1.3% | 1 | 0.5% | 0 | 0.0% |
| **Supplementary Table S1d: Frequency of participant responses for urination module questions by random split halves and life stage categories. *(Continued).***  ***Question:*** ***How often have you experienced any of the following in the previous 30 days when going to urinate?*** | | | | | | | | | | | | | | | | | | | | | | |
|  | ***Full Sample*** | | ***Sub-Sample N_1_*** | | | | | | | | | | ***Sub-Sample N_2_*** | | | | | | | | | |
|  | All  N=1408 | | All  N_1_=703 | | 1. Unmarried (UM)  n=166 | | 2. Recently Married (RM) n=162 | | 3. Married (M)  n=185 | | 4. Over 49 (OW)  n=190 | | All  N_2_=705 | | 1. Unmarried (UM)  n=175 | | 2. Recently Married (RM) n=158 | | 3. Married (M)  n=210 | | 4. Over 49 (OW)  n=162 | |
|  |  |  |  |  |  |  |  |  |  |  |  |  |  |  |  |  |  |  |  |  |  |  |
| **u28 Withheld water to control urge to urinate** | | | | | | | | | | | | | | | | | | | | | | |
| Never | 1278 | 90.8% | 647 | 92.0% | 147 | 88.6% | 151 | 93.2% | 174 | 94.1% | 175 | 92.1% | 631 | 89.5% | 154 | 88.0% | 138 | 87.3% | 194 | 92.4% | 145 | 89.5% |
| Sometimes | 101 | 7.2% | 45 | 6.4% | 16 | 9.6% | 8 | 4.9% | 11 | 5.9% | 10 | 5.3% | 56 | 7.9% | 13 | 7.4% | 16 | 10.1% | 14 | 6.7% | 13 | 8.0% |
| Often | 12 | 0.9% | 6 | 0.9% | 2 | 1.2% | 2 | 1.2% | 0 | 0.0% | 2 | 1.1% | 6 | 0.9% | 1 | 0.6% | 2 | 1.3% | 2 | 1.0% | 1 | 0.6% |
| Always | 17 | 1.2% | 5 | 0.7% | 1 | 0.6% | 1 | 0.6% | 0 | 0.0% | 3 | 1.6% | 12 | 1.7% | 7 | 4.0% | 2 | 1.3% | 0 | 0.0% | 3 | 1.9% |
| **u29 Had to suppress when workload was high^2^** | | | | | | | | | | | | | | | | | | | | | | |
| Never | 835 | 59.4% | 423 | 60.2% | 91 | 54.8% | 92 | 56.8% | 99 | 53.5% | 141 | 74.2% | 412 | 58.6% | 94 | 53.7% | 92 | 58.2% | 113 | 54.1% | 113 | 70.2% |
| Sometimes | 531 | 37.8% | 259 | 36.8% | 73 | 44.0% | 64 | 39.5% | 75 | 40.5% | 47 | 24.7% | 272 | 38.7% | 75 | 42.9% | 59 | 37.3% | 92 | 44.0% | 46 | 28.6% |
| Often | 36 | 2.6% | 20 | 2.8% | 2 | 1.2% | 6 | 3.7% | 10 | 5.4% | 2 | 1.1% | 16 | 2.3% | 4 | 2.3% | 6 | 3.8% | 4 | 1.9% | 2 | 1.2% |
| Always | 4 | 0.3% | 1 | 0.1% | 0 | 0.0% | 0 | 0.0% | 1 | 0.5% | 0 | 0.0% | 3 | 0.4% | 2 | 1.1% | 1 | 0.6% | 0 | 0.0% | 0 | 0.0% |
| **u30 Had men or boys harm or harass me when going to urinate** | | | | | | | | | | | | | | | | | | | | | | |
| Never | 1407 | 99.9% | 703 | 100% | 166 | 100% | 162 | 100% | 185 | 100% | 190 | 100% | 704 | 99.9% | 175 | 100% | 157 | 99.4% | 210 | 100% | 162 | 100% |
| Sometimes | 1 | 0.1% | 0 | 0.0% | 0 | 0.0% | 0 | 0.0% | 0 | 0.0% | 0 | 0.0% | 1 | 0.1% | 0 | 0.0% | 1 | 0.6% | 0 | 0.0% | 0 | 0.0% |
| Often | 0 | 0.0% | 0 | 0.0% | 0 | 0.0% | 0 | 0.0% | 0 | 0.0% | 0 | 0.0% | 0 | 0.0% | 0 | 0.0% | 0 | 0.0% | 0 | 0.0% | 0 | 0.0% |
| Always | 0 | 0.0% | 0 | 0.0% | 0 | 0.0% | 0 | 0.0% | 0 | 0.0% | 0 | 0.0% | 0 | 0.0% | 0 | 0.0% | 0 | 0.0% | 0 | 0.0% | 0 | 0.0% |
| **u31 Worried others would get upset if asked to accompany for urination** | | | | | | | | | | | | | | | | | | | | | | |
| Never | 1265 | 89.8% | 638 | 90.8% | 134 | 80.7% | 141 | 87.0% | 177 | 95.7% | 186 | 97.9% | 627 | 88.9% | 154 | 88.0% | 128 | 81.0% | 190 | 90.5% | 155 | 95.7% |
| Sometimes | 121 | 8.6% | 57 | 8.1% | 29 | 17.5% | 18 | 11.1% | 8 | 4.3% | 2 | 1.1% | 64 | 9.1% | 18 | 10.3% | 21 | 13.3% | 19 | 9.0% | 6 | 3.7% |
| Often | 13 | 0.9% | 5 | 0.7% | 1 | 0.6% | 3 | 1.9% | 0 | 0.0% | 1 | 0.5% | 8 | 1.1% | 2 | 1.1% | 4 | 2.5% | 1 | 0.5% | 1 | 0.6% |
| Always | 9 | 0.6% | 3 | 0.4% | 2 | 1.2% | 0 | 0.0% | 0 | 0.0% | 1 | 0.5% | 6 | 0.9% | 1 | 0.6% | 5 | 3.2% | 0 | 0.0% | 0 | 0.0% |
| **u32 Had to suppress when I got an urge at night** | | | | | | | | | | | | | | | | | | | | | | |
| Never | 999 | 71.0% | 506 | 72.0% | 103 | 62.0% | 111 | 68.5% | 138 | 74.6% | 154 | 81.1% | 493 | 69.9% | 124 | 70.9% | 98 | 62.0% | 148 | 70.5% | 123 | 75.9% |
| Sometimes | 332 | 23.6% | 160 | 22.8% | 51 | 30.7% | 37 | 22.8% | 38 | 20.5% | 34 | 17.9% | 172 | 24.4% | 37 | 21.1% | 46 | 29.1% | 56 | 26.7% | 33 | 20.4% |
| Often | 64 | 4.5% | 34 | 4.8% | 9 | 5.4% | 14 | 8.6% | 9 | 4.9% | 2 | 1.1% | 30 | 4.3% | 11 | 6.3% | 10 | 6.3% | 5 | 2.4% | 4 | 2.5% |
| Always | 13 | 0.9% | 3 | 0.4% | 3 | 1.8% | 0 | 0.0% | 0 | 0.0% | 0 | 0.0% | 10 | 1.4% | 3 | 1.7% | 4 | 2.5% | 1 | 0.5% | 2 | 1.2% |
| 1.Chi-Square significant differences: U01, Stage C p=0.0285; U02, Stage A p=0.0.0036; U10, By overall split half group p=0.0217; U18, Stage C p=0.0436.  2. Missings: U08, 1 missing (N_1_, stage D); U26: 1 missing (N_1_, stage A); U29: 2 missing (N_2_, stage C & stage D) | | | | | | | | | | | | | | | | | | | | | | |

| **Supplementary Table S2a: Frequency of participant responses for defecation module questions by random split halves and life stage categories.**  ***Question:*** ***How often have you experienced any of the following in the previous 30 days when going to defecate?*** | | | | | | | | | | | | | | | | | | | | | | |
| --- | --- | --- | --- | --- | --- | --- | --- | --- | --- | --- | --- | --- | --- | --- | --- | --- | --- | --- | --- | --- | --- | --- |
|  | ***Full Sample*** | | ***Sub-Sample N_1_*** | | | | | | | | | | ***Sub-Sample N_2_*** | | | | | | | | | |
|  | All  N=1408 | | All  N_1_=703 | | 1. Unmarried (UM)  n=166 | | 2. Recently Married (RM) n=162 | | 3. Married (M)  n=185 | | 4. Over 49 (OW)  n=190 | | All  N_2_=705 | | 1. Unmarried (UM)  n=175 | | 2. Recently Married (RM) n=158 | | 3. Married (M)  n=210 | | 4. Over 49 (OW)  n=162 | |
|  |  |  |  |  |  |  |  |  |  |  |  |  |  |  |  |  |  |  |  |  |  |  |
| **d01 Worried about not having a toilet to defecate^1^** | | | | | | | | | | | | | | | | | | | | | | |
| Never | 543 | 38.6% | 264 | 37.6% | 49 | 29.5% | 80 | 49.4% | 59 | 32.1% | 76 | 40.0% | 279 | 39.6% | 57 | 32.6% | 81 | 51.3% | 71 | 33.8% | 70 | 43.2% |
| Sometimes | 33 | 2.3% | 19 | 2.7% | 6 | 3.6% | 1 | 0.6% | 7 | 3.8% | 5 | 2.6% | 14 | 2.0% | 3 | 1.7% | 2 | 1.3% | 3 | 1.4% | 6 | 3.7% |
| Often | 78 | 5.5% | 37 | 5.3% | 15 | 9.0% | 7 | 4.3% | 11 | 6.0% | 4 | 2.1% | 41 | 5.8% | 12 | 6.9% | 6 | 3.8% | 14 | 6.7% | 9 | 5.6% |
| Always | 753 | 53.5% | 382 | 54.4% | 96 | 57.8% | 74 | 45.7% | 107 | 58.2% | 105 | 55.3% | 371 | 52.6% | 103 | 58.9% | 69 | 43.7% | 122 | 58.1% | 77 | 47.5% |
| **d02 Had to go far to defecate** | | | | | | | | | | | | | | | | | | | | | | |
| Never | 582 | 41.3% | 282 | 40.1% | 52 | 31.3% | 94 | 58.0% | 59 | 31.9% | 77 | 40.5% | 300 | 42.6% | 60 | 34.3% | 92 | 58.2% | 79 | 37.6% | 69 | 42.6% |
| Sometimes | 68 | 4.8% | 31 | 4.4% | 9 | 5.4% | 3 | 1.9% | 11 | 5.9% | 8 | 4.2% | 37 | 5.2% | 6 | 3.4% | 5 | 3.2% | 13 | 6.2% | 13 | 8.0% |
| Often | 135 | 9.6% | 72 | 10.2% | 25 | 15.1% | 7 | 4.3% | 17 | 9.2% | 23 | 12.1% | 63 | 8.9% | 13 | 7.4% | 4 | 2.5% | 27 | 12.9% | 19 | 11.7% |
| Always | 623 | 44.2% | 318 | 45.2% | 80 | 48.2% | 58 | 35.8% | 98 | 53.0% | 82 | 43.2% | 305 | 43.3% | 96 | 54.9% | 57 | 36.1% | 91 | 43.3% | 61 | 37.7% |
| **d03 Defecation process/activity of defecation took a long time to complete** | | | | | | | | | | | | | | | | | | | | | | |
| Never | 596 | 42.3% | 297 | 42.2% | 57 | 34.3% | 90 | 55.6% | 69 | 37.3% | 81 | 42.6% | 299 | 42.4% | 63 | 36.0% | 85 | 53.8% | 85 | 40.5% | 66 | 40.7% |
| Sometimes | 133 | 9.4% | 72 | 10.2% | 27 | 16.3% | 10 | 6.2% | 20 | 10.8% | 15 | 7.9% | 61 | 8.7% | 18 | 10.3% | 10 | 6.3% | 14 | 6.7% | 19 | 11.7% |
| Often | 211 | 15.0% | 106 | 15.1% | 24 | 14.5% | 20 | 12.3% | 27 | 14.6% | 35 | 18.4% | 105 | 14.9% | 26 | 14.9% | 20 | 12.7% | 36 | 17.1% | 23 | 14.2% |
| Always | 468 | 33.2% | 228 | 32.4% | 58 | 34.9% | 42 | 25.9% | 69 | 37.3% | 59 | 31.1% | 240 | 34.0% | 68 | 38.9% | 43 | 27.2% | 75 | 35.7% | 54 | 33.3% |
| **d04 Experienced pain during defecation** | | | | | | | | | | | | | | | | | | | | | | |
| Never | 1097 | 77.5% | 536 | 76.2% | 144 | 86.7% | 124 | 76.5% | 138 | 74.6% | 130 | 68.4% | 561 | 79.6% | 154 | 88.0% | 125 | 79.1% | 166 | 79.0% | 116 | 71.6% |
| Sometimes | 166 | 11.7% | 86 | 12.2% | 16 | 9.6% | 15 | 9.3% | 26 | 14.1% | 29 | 15.3% | 80 | 11.3% | 12 | 6.9% | 18 | 11.4% | 25 | 11.9% | 25 | 15.4% |
| Often | 41 | 2.9% | 25 | 3.6% | 3 | 1.8% | 8 | 4.9% | 7 | 3.8% | 7 | 3.7% | 16 | 2.3% | 2 | 1.1% | 3 | 1.9% | 5 | 2.4% | 6 | 3.7% |
| Always | 104 | 7.3% | 56 | 8.0% | 3 | 1.8% | 15 | 9.3% | 14 | 7.6% | 24 | 12.6% | 48 | 6.8% | 7 | 4.0% | 12 | 7.6% | 14 | 6.7% | 15 | 9.3% |
| **d05 Had difficulty finding a clean place to defecate** | | | | | | | | | | | | | | | | | | | | | | |
| Never | 724 | 51.4% | 360 | 51.2% | 69 | 41.6% | 98 | 60.5% | 93 | 50.3% | 100 | 52.6% | 364 | 51.6% | 80 | 45.7% | 95 | 60.1% | 100 | 47.6% | 89 | 54.9% |
| Sometimes | 195 | 13.8% | 101 | 14.4% | 27 | 16.3% | 16 | 9.9% | 28 | 15.1% | 30 | 15.8% | 94 | 13.3% | 24 | 13.7% | 16 | 10.1% | 30 | 14.3% | 24 | 14.8% |
| Often | 125 | 8.9% | 61 | 8.7% | 15 | 9.0% | 6 | 3.7% | 18 | 9.7% | 22 | 11.6% | 64 | 9.1% | 16 | 9.1% | 12 | 7.6% | 23 | 11.0% | 13 | 8.0% |
| Always | 364 | 25.9% | 181 | 25.7% | 55 | 33.1% | 42 | 25.9% | 46 | 24.9% | 38 | 20.0% | 183 | 26.0% | 55 | 31.4% | 35 | 22.2% | 57 | 27.1% | 36 | 22.2% |
| **d06 Could not access preferred location** | | | | | | | | | | | | | | | | | | | | | | |
| Never | 1019 | 72.4% | 516 | 73.4% | 114 | 68.7% | 127 | 78.4% | 135 | 73.0% | 140 | 73.7% | 503 | 71.3% | 119 | 68.0% | 119 | 75.3% | 145 | 69.0% | 120 | 74.1% |
| Sometimes | 259 | 18.4% | 130 | 18.5% | 40 | 24.1% | 22 | 13.6% | 32 | 17.3% | 36 | 18.9% | 129 | 18.3% | 30 | 17.1% | 18 | 11.4% | 47 | 22.4% | 34 | 21.0% |
| Often | 20 | 1.4% | 8 | 1.1% | 0 | 0.0% | 2 | 1.2% | 3 | 1.6% | 3 | 1.6% | 12 | 1.7% | 3 | 1.7% | 4 | 2.5% | 3 | 1.4% | 2 | 1.2% |
| Always | 110 | 7.8% | 49 | 7.0% | 12 | 7.2% | 11 | 6.8% | 15 | 8.1% | 11 | 5.8% | 63 | 8.9% | 23 | 13.1% | 17 | 10.8% | 15 | 7.1% | 6 | 3.7% |
| **d07 Worried I would fall when going to defecate** | | | | | | | | | | | | | | | | | | | | | | |
| Never | 1195 | 84.9% | 596 | 84.8% | 155 | 93.4% | 147 | 90.7% | 175 | 94.6% | 119 | 62.6% | 599 | 85.0% | 166 | 94.9% | 145 | 91.8% | 189 | 90.0% | 99 | 61.1% |
| Sometimes | 110 | 7.8% | 54 | 7.7% | 8 | 4.8% | 9 | 5.6% | 7 | 3.8% | 30 | 15.8% | 56 | 7.9% | 6 | 3.4% | 9 | 5.7% | 13 | 6.2% | 28 | 17.3% |
| Often | 49 | 3.5% | 25 | 3.6% | 3 | 1.8% | 5 | 3.1% | 1 | 0.5% | 16 | 8.4% | 24 | 3.4% | 1 | 0.6% | 3 | 1.9% | 6 | 2.9% | 14 | 8.6% |
| Always | 54 | 3.8% | 28 | 4.0% | 0 | 0.0% | 1 | 0.6% | 2 | 1.1% | 25 | 13.2% | 26 | 3.7% | 2 | 1.1% | 1 | 0.6% | 2 | 1.0% | 21 | 13.0% |
| **d08 Worried that people would see me defecating** | | | | | | | | | | | | | | | | | | | | | | |
| Never | 869 | 61.7% | 435 | 61.9% | 78 | 47.0% | 105 | 64.8% | 108 | 58.4% | 144 | 75.8% | 434 | 61.6% | 85 | 48.6% | 98 | 62.0% | 119 | 56.7% | 132 | 81.5% |
| Sometimes | 224 | 15.9% | 112 | 15.9% | 27 | 16.3% | 28 | 17.3% | 36 | 19.5% | 21 | 11.1% | 112 | 15.9% | 31 | 17.7% | 22 | 13.9% | 38 | 18.1% | 21 | 13.0% |
| Often | 118 | 8.4% | 64 | 9.1% | 27 | 16.3% | 9 | 5.6% | 17 | 9.2% | 11 | 5.8% | 54 | 7.7% | 20 | 11.4% | 8 | 5.1% | 23 | 11.0% | 3 | 1.9% |
| Always | 197 | 14.0% | 92 | 13.1% | 34 | 20.5% | 20 | 12.3% | 24 | 13.0% | 14 | 7.4% | 105 | 14.9% | 39 | 22.3% | 30 | 19.0% | 30 | 14.3% | 6 | 3.7% |
| **d09 Had to suppress urge when workload was high** | | | | | | | | | | | | | | | | | | | | | | |
| Never | 816 | 58.0% | 410 | 58.3% | 89 | 53.6% | 97 | 59.9% | 94 | 50.8% | 130 | 68.4% | 406 | 57.6% | 98 | 56.0% | 89 | 56.3% | 105 | 50.0% | 114 | 70.4% |
| Sometimes | 522 | 37.1% | 259 | 36.8% | 69 | 41.6% | 56 | 34.6% | 81 | 43.8% | 53 | 27.9% | 263 | 37.3% | 67 | 38.3% | 60 | 38.0% | 89 | 42.4% | 47 | 29.0% |
| Often | 62 | 4.4% | 30 | 4.3% | 8 | 4.8% | 7 | 4.3% | 9 | 4.9% | 6 | 3.2% | 32 | 4.5% | 9 | 5.1% | 8 | 5.1% | 14 | 6.7% | 1 | 0.6% |
| Always | 8 | 0.6% | 4 | 0.6% | 0 | 0.0% | 2 | 1.2% | 1 | 0.5% | 1 | 0.5% | 4 | 0.6% | 1 | 0.6% | 1 | 0.6% | 2 | 1.0% | 0 | 0.0% |
| **Supplementary Table S2b: Frequency of participant responses for defecation module questions by random split halves and life stage categories. *(Continued).***  ***Question:*** ***How often have you experienced any of the following in the previous 30 days when going to defecate?*** | | | | | | | | | | | | | | | | | | | | | | |
|  | ***Full Sample*** | | ***Sub-Sample N_1_*** | | | | | | | | | | ***Sub-Sample N_2_*** | | | | | | | | | |
|  | All  N=1408 | | All  N_1_=703 | | 1. Unmarried (UM)  n=166 | | 2. Recently Married (RM) n=162 | | 3. Married (M)  n=185 | | 4. Over 49 (OW)  n=190 | | All  N_2_=705 | | 1. Unmarried (UM)  n=175 | | 2. Recently Married (RM) n=158 | | 3. Married (M)  n=210 | | 4. Over 49 (OW)  n=162 | |
|  |  |  |  |  |  |  |  |  |  |  |  |  |  |  |  |  |  |  |  |  |  |  |
| **d10 Felt scared defecating in the dark at night** | | | | | | | | | | | | | | | | | | | | | | |
| Never | 579 | 41.1% | 295 | 42.0% | 43 | 25.9% | 61 | 37.7% | 78 | 42.2% | 113 | 59.5% | 284 | 40.3% | 49 | 28.0% | 48 | 30.4% | 85 | 40.5% | 102 | 63.0% |
| Sometimes | 307 | 21.8% | 152 | 21.6% | 44 | 26.5% | 24 | 14.8% | 50 | 27.0% | 34 | 17.9% | 155 | 22.0% | 36 | 20.6% | 36 | 22.8% | 56 | 26.7% | 27 | 16.7% |
| Often | 187 | 13.3% | 91 | 12.9% | 27 | 16.3% | 26 | 16.0% | 20 | 10.8% | 18 | 9.5% | 96 | 13.6% | 33 | 18.9% | 25 | 15.8% | 23 | 11.0% | 15 | 9.3% |
| Always | 335 | 23.8% | 165 | 23.5% | 52 | 31.3% | 51 | 31.5% | 37 | 20.0% | 25 | 13.2% | 170 | 24.1% | 57 | 32.6% | 49 | 31.0% | 46 | 21.9% | 18 | 11.1% |
| **d11 Worried people would talk about me if they saw me^2^** | | | | | | | | | | | | | | | | | | | | | | |
| Never | 1161 | 82.5% | 583 | 82.9% | 121 | 72.9% | 126 | 77.8% | 160 | 86.5% | 176 | 92.6% | 578 | 82.0% | 138 | 78.9% | 130 | 82.3% | 161 | 76.7% | 149 | 92.0% |
| Sometimes | 83 | 5.9% | 33 | 4.7% | 9 | 5.4% | 12 | 7.4% | 8 | 4.3% | 4 | 2.1% | 50 | 7.1% | 18 | 10.3% | 10 | 6.3% | 14 | 6.7% | 8 | 4.9% |
| Often | 89 | 6.3% | 48 | 6.8% | 22 | 13.3% | 9 | 5.6% | 10 | 5.4% | 7 | 3.7% | 41 | 5.8% | 10 | 5.7% | 8 | 5.1% | 20 | 9.5% | 3 | 1.9% |
| Always | 75 | 5.3% | 39 | 5.5% | 14 | 8.4% | 15 | 9.3% | 7 | 3.8% | 3 | 1.6% | 36 | 5.1% | 9 | 5.1% | 10 | 6.3% | 15 | 7.1% | 2 | 1.2% |
| **d12 Feared I would be harmed by animals or insects when I went to defecate** | | | | | | | | | | | | | | | | | | | | | | |
| Never | 1089 | 77.3% | 541 | 77.0% | 119 | 71.7% | 127 | 78.4% | 142 | 76.8% | 153 | 80.5% | 548 | 77.7% | 131 | 74.9% | 124 | 78.5% | 155 | 73.8% | 138 | 85.2% |
| Sometimes | 142 | 10.1% | 73 | 10.4% | 21 | 12.7% | 20 | 12.3% | 16 | 8.6% | 16 | 8.4% | 69 | 9.8% | 17 | 9.7% | 13 | 8.2% | 24 | 11.4% | 15 | 9.3% |
| Often | 36 | 2.6% | 16 | 2.3% | 4 | 2.4% | 2 | 1.2% | 5 | 2.7% | 5 | 2.6% | 20 | 2.8% | 5 | 2.9% | 6 | 3.8% | 6 | 2.9% | 3 | 1.9% |
| Always | 141 | 10.0% | 73 | 10.4% | 22 | 13.3% | 13 | 8.0% | 22 | 11.9% | 16 | 8.4% | 68 | 9.6% | 22 | 12.6% | 15 | 9.5% | 25 | 11.9% | 6 | 3.7% |
| **d13 Got wounds on my feet when walking to defecate** | | | | | | | | | | | | | | | | | | | | | | |
| Never | 1402 | 99.6% | 702 | 99.9% | 166 | 100% | 162 | 100% | 184 | 99.5% | 190 | 100% | 700 | 99.3% | 173 | 98.9% | 157 | 99.4% | 209 | 99.5% | 161 | 99.4% |
| Sometimes | 3 | 0.2% | 0 | 0.0% | 0 | 0.0% | 0 | 0.0% | 0 | 0.0% | 0 | 0.0% | 3 | 0.4% | 1 | 0.6% | 0 | 0.0% | 1 | 0.5% | 1 | 0.6% |
| Often | 2 | 0.1% | 1 | 0.1% | 0 | 0.0% | 0 | 0.0% | 1 | 0.5% | 0 | 0.0% | 1 | 0.1% | 0 | 0.0% | 1 | 0.6% | 0 | 0.0% | 0 | 0.0% |
| Always | 1 | 0.1% | 0 | 0.0% | 0 | 0.0% | 0 | 0.0% | 0 | 0.0% | 0 | 0.0% | 1 | 0.1% | 1 | 0.6% | 0 | 0.0% | 0 | 0.0% | 0 | 0.0% |
| **d14 Had to do extra work washing clothes because of dirty conditions where defecating^2^** | | | | | | | | | | | | | | | | | | | | | | |
| Never | 819 | 58.2% | 429 | 61.0% | 88 | 53.0% | 104 | 64.2% | 113 | 61.1% | 124 | 65.3% | 390 | 55.3% | 92 | 52.6% | 91 | 57.6% | 103 | 49.0% | 104 | 64.2% |
| Sometimes | 163 | 11.6% | 82 | 11.7% | 38 | 22.9% | 15 | 9.3% | 12 | 6.5% | 17 | 8.9% | 81 | 11.5% | 26 | 14.9% | 14 | 8.9% | 24 | 11.4% | 17 | 10.5% |
| Often | 97 | 6.9% | 33 | 4.7% | 13 | 7.8% | 8 | 4.9% | 7 | 3.8% | 5 | 2.6% | 64 | 9.1% | 10 | 5.7% | 10 | 6.3% | 31 | 14.8% | 13 | 8.0% |
| Always | 329 | 23.4% | 159 | 22.6% | 27 | 16.3% | 35 | 21.6% | 53 | 28.6% | 44 | 23.2% | 170 | 24.1% | 47 | 26.9% | 43 | 27.2% | 52 | 24.8% | 28 | 17.3% |
| **d15 Worried about getting an infection when going to defecate** | | | | | | | | | | | | | | | | | | | | | | |
| Never | 862 | 61.2% | 442 | 62.9% | 78 | 47.0% | 101 | 62.3% | 118 | 63.8% | 145 | 76.3% | 420 | 59.6% | 82 | 46.9% | 94 | 59.5% | 120 | 57.1% | 124 | 76.5% |
| Sometimes | 168 | 11.9% | 83 | 11.8% | 30 | 18.1% | 15 | 9.3% | 23 | 12.4% | 15 | 7.9% | 85 | 12.1% | 28 | 16.0% | 17 | 10.8% | 25 | 11.9% | 15 | 9.3% |
| Often | 89 | 6.3% | 37 | 5.3% | 18 | 10.8% | 7 | 4.3% | 6 | 3.2% | 6 | 3.2% | 52 | 7.4% | 14 | 8.0% | 10 | 6.3% | 20 | 9.5% | 8 | 4.9% |
| Always | 289 | 20.5% | 141 | 20.1% | 40 | 24.1% | 39 | 24.1% | 38 | 20.5% | 24 | 12.6% | 148 | 21.0% | 51 | 29.1% | 37 | 23.4% | 45 | 21.4% | 15 | 9.3% |
| **d16 Had people tease me if they saw me defecating** | | | | | | | | | | | | | | | | | | | | | | |
| Never | 1392 | 98.9% | 696 | 99.0% | 161 | 97.0% | 161 | 99.4% | 184 | 99.5% | 190 | 100% | 696 | 98.7% | 173 | 98.9% | 156 | 98.7% | 207 | 98.6% | 160 | 98.8% |
| Sometimes | 11 | 0.8% | 5 | 0.7% | 3 | 1.8% | 1 | 0.6% | 1 | 0.5% | 0 | 0.0% | 6 | 0.9% | 2 | 1.1% | 0 | 0.0% | 3 | 1.4% | 1 | 0.6% |
| Often | 2 | 0.1% | 1 | 0.1% | 1 | 0.6% | 0 | 0.0% | 0 | 0.0% | 0 | 0.0% | 1 | 0.1% | 0 | 0.0% | 1 | 0.6% | 0 | 0.0% | 0 | 0.0% |
| Always | 3 | 0.2% | 1 | 0.1% | 1 | 0.6% | 0 | 0.0% | 0 | 0.0% | 0 | 0.0% | 2 | 0.3% | 0 | 0.0% | 1 | 0.6% | 0 | 0.0% | 1 | 0.6% |
| **d17 Had difficulty or pain squatting for defecation** | | | | | | | | | | | | | | | | | | | | | | |
| Never | 933 | 66.3% | 452 | 64.3% | 129 | 77.7% | 117 | 72.2% | 143 | 73.3% | 125 | 65.8% | 481 | 68.2% | 146 | 83.4% | 125 | 79.1% | 150 | 71.4% | 60 | 37.0% |
| Sometimes | 253 | 18.0% | 133 | 18.9% | 26 | 15.7% | 28 | 17.3% | 24 | 12.3% | 39 | 20.5% | 120 | 17.0% | 19 | 10.9% | 20 | 12.7% | 41 | 19.5% | 40 | 24.7% |
| Often | 68 | 4.8% | 32 | 4.6% | 3 | 1.8% | 4 | 2.5% | 9 | 4.6% | 15 | 7.9% | 36 | 5.1% | 3 | 1.7% | 5 | 3.2% | 7 | 3.3% | 21 | 13.0% |
| Always | 154 | 10.9% | 86 | 12.2% | 8 | 4.8% | 13 | 8.0% | 9 | 4.6% | 11 | 5.8% | 68 | 9.6% | 7 | 4.0% | 8 | 5.1% | 12 | 5.7% | 41 | 25.3% |
| **d18 Felt scared of ghosts when I went to defecate at night^2^** | | | | | | | | | | | | | | | | | | | | | | |
| Never | 576 | 40.9% | 285 | 40.5% | 41 | 24.7% | 55 | 34.0% | 75 | 40.5% | 114 | 60.0% | 291 | 41.3% | 46 | 26.3% | 41 | 25.9% | 95 | 45.2% | 109 | 67.3% |
| Sometimes | 328 | 23.3% | 170 | 24.2% | 48 | 28.9% | 31 | 19.1% | 54 | 29.2% | 37 | 19.5% | 158 | 22.4% | 36 | 20.6% | 42 | 26.6% | 52 | 24.8% | 28 | 17.3% |
| Often | 196 | 13.9% | 102 | 14.5% | 38 | 22.9% | 29 | 17.9% | 21 | 11.4% | 14 | 7.4% | 94 | 13.3% | 29 | 16.6% | 30 | 19.0% | 24 | 11.4% | 11 | 6.8% |
| Always | 308 | 21.9% | 146 | 20.8% | 39 | 23.5% | 47 | 29.0% | 35 | 18.9% | 25 | 13.2% | 162 | 23.0% | 64 | 36.6% | 45 | 28.5% | 39 | 18.6% | 14 | 8.6% |
| **Supplementary Table S2c: Frequency of participant responses for defecation module questions by random split halves and life stage categories. *(Continued).***  ***Question:*** ***How often have you experienced any of the following in the previous 30 days when going to defecate?*** | | | | | | | | | | | | | | | | | | | | | | |
|  | ***Full Sample*** | | ***Sub-Sample N_1_*** | | | | | | | | | | ***Sub-Sample N_2_*** | | | | | | | | | |
|  | All  N=1408 | | All  N_1_=703 | | 1. Unmarried (UM)  n=166 | | 2. Recently Married (RM) n=162 | | 3. Married (M)  n=185 | | 4. Over 49 (OW)  n=190 | | All  N_2_=705 | | 1. Unmarried (UM)  n=175 | | 2. Recently Married (RM) n=158 | | 3. Married (M)  n=210 | | 4. Over 49 (OW)  n=162 | |
|  |  |  |  |  |  |  |  |  |  |  |  |  |  |  |  |  |  |  |  |  |  |  |
| **d19 Had difficulty walking to defecation place** | | | | | | | | | | | | | | | | | | | | | | |
| Never | 1128 | 80.1% | 563 | 80.1% | 135 | 81.3% | 138 | 85.2% | 157 | 84.9% | 133 | 70.0% | 565 | 80.1% | 144 | 82.3% | 139 | 88.0% | 171 | 81.4% | 111 | 68.5% |
| Sometimes | 173 | 12.3% | 85 | 12.1% | 22 | 13.3% | 18 | 11.1% | 18 | 9.7% | 27 | 14.2% | 88 | 12.5% | 16 | 9.1% | 14 | 8.9% | 30 | 14.3% | 28 | 17.3% |
| Often | 48 | 3.4% | 23 | 3.3% | 4 | 2.4% | 3 | 1.9% | 8 | 4.3% | 8 | 4.2% | 25 | 3.5% | 5 | 2.9% | 3 | 1.9% | 7 | 3.3% | 10 | 6.2% |
| Always | 59 | 4.2% | 32 | 4.6% | 5 | 3.0% | 3 | 1.9% | 2 | 1.1% | 22 | 11.6% | 27 | 3.8% | 10 | 5.7% | 2 | 1.3% | 2 | 1.0% | 13 | 8.0% |
| **d20 Had to find someone to look after my work so I could defecate** | | | | | | | | | | | | | | | | | | | | | | |
| Never | 838 | 59.5% | 416 | 59.2% | 89 | 53.6% | 78 | 48.1% | 106 | 57.3% | 143 | 75.3% | 422 | 59.9% | 109 | 62.3% | 74 | 46.8% | 116 | 55.2% | 123 | 75.9% |
| Sometimes | 465 | 33.0% | 239 | 34.0% | 68 | 41.0% | 63 | 38.9% | 67 | 36.2% | 41 | 21.6% | 226 | 32.1% | 56 | 32.0% | 63 | 39.9% | 71 | 33.8% | 36 | 22.2% |
| Often | 86 | 6.1% | 41 | 5.8% | 9 | 5.4% | 16 | 9.9% | 11 | 5.9% | 5 | 2.6% | 45 | 6.4% | 8 | 4.6% | 15 | 9.5% | 19 | 9.0% | 3 | 1.9% |
| Always | 19 | 1.3% | 7 | 1.0% | 0 | 0.0% | 5 | 3.1% | 1 | 0.5% | 1 | 0.5% | 12 | 1.7% | 2 | 1.1% | 6 | 3.8% | 4 | 1.9% | 0 | 0.0% |
| **d21 Had difficulty accessing water for defecation^2^** | | | | | | | | | | | | | | | | | | | | | | |
| Never | 1289 | 91.5% | 643 | 91.5% | 148 | 89.2% | 151 | 93.2% | 164 | 88.6% | 180 | 94.7% | 646 | 91.6% | 157 | 89.7% | 139 | 88.0% | 194 | 92.4% | 156 | 96.3% |
| Sometimes | 51 | 3.6% | 30 | 4.3% | 11 | 6.6% | 6 | 3.7% | 6 | 3.2% | 7 | 3.7% | 21 | 3.0% | 4 | 2.3% | 5 | 3.2% | 8 | 3.8% | 4 | 2.5% |
| Often | 15 | 1.1% | 10 | 1.4% | 3 | 1.8% | 1 | 0.6% | 6 | 3.2% | 0 | 0.0% | 5 | 0.7% | 1 | 0.6% | 0 | 0.0% | 4 | 1.9% | 0 | 0.0% |
| Always | 53 | 3.8% | 20 | 2.8% | 4 | 2.4% | 4 | 2.5% | 9 | 4.9% | 3 | 1.6% | 33 | 4.7% | 13 | 7.4% | 14 | 8.9% | 4 | 1.9% | 2 | 1.2% |
| **d22 Changing and washing clothes used only for defecation increased workload^2^** | | | | | | | | | | | | | | | | | | | | | | |
| Never | 632 | 44.9% | 330 | 46.9% | 72 | 43.4% | 67 | 41.4% | 78 | 42.2% | 113 | 59.5% | 302 | 42.8% | 78 | 44.6% | 63 | 39.9% | 78 | 37.1% | 83 | 51.2% |
| Sometimes | 180 | 12.8% | 84 | 11.9% | 33 | 19.9% | 16 | 9.9% | 20 | 10.8% | 15 | 7.9% | 96 | 13.6% | 19 | 10.9% | 22 | 13.9% | 26 | 12.4% | 29 | 17.9% |
| Often | 85 | 6.0% | 38 | 5.4% | 16 | 9.6% | 8 | 4.9% | 10 | 5.4% | 4 | 2.1% | 47 | 6.7% | 13 | 7.4% | 12 | 7.6% | 18 | 8.6% | 4 | 2.5% |
| Always | 511 | 36.3% | 251 | 35.7% | 45 | 27.1% | 71 | 43.8% | 77 | 41.6% | 58 | 30.5% | 260 | 36.9% | 65 | 37.1% | 61 | 38.6% | 88 | 41.9% | 46 | 28.4% |
| **d23 Had difficulty cleaning/washing myself after defecation^2^** | | | | | | | | | | | | | | | | | | | | | | |
| Never | 1297 | 92.1% | 648 | 92.2% | 150 | 90.4% | 153 | 94.4% | 167 | 90.3% | 178 | 93.7% | 649 | 92.1% | 157 | 89.7% | 139 | 88.0% | 197 | 93.8% | 156 | 96.3% |
| Sometimes | 52 | 3.7% | 27 | 3.8% | 9 | 5.4% | 4 | 2.5% | 12 | 6.5% | 2 | 1.1% | 25 | 3.5% | 5 | 2.9% | 6 | 3.8% | 9 | 4.3% | 5 | 3.1% |
| Often | 13 | 0.9% | 8 | 1.1% | 3 | 1.8% | 0 | 0.0% | 1 | 0.5% | 4 | 2.1% | 5 | 0.7% | 2 | 1.1% | 1 | 0.6% | 2 | 1.0% | 0 | 0.0% |
| Always | 46 | 3.3% | 20 | 2.8% | 4 | 2.4% | 5 | 3.1% | 5 | 2.7% | 6 | 3.2% | 26 | 3.7% | 11 | 6.3% | 12 | 7.6% | 2 | 1.0% | 1 | 0.6% |
| **d24 Had to suppress urge to defecate because people were around** | | | | | | | | | | | | | | | | | | | | | | |
| Never | 743 | 52.8% | 379 | 53.9% | 68 | 41.0% | 100 | 61.7% | 84 | 45.4% | 127 | 66.8% | 364 | 51.6% | 63 | 36.0% | 92 | 58.2% | 105 | 50.0% | 104 | 64.2% |
| Sometimes | 551 | 39.1% | 275 | 39.1% | 79 | 47.6% | 51 | 31.5% | 88 | 47.6% | 57 | 30.0% | 276 | 39.1% | 84 | 48.0% | 53 | 33.5% | 85 | 40.5% | 54 | 33.3% |
| Often | 92 | 6.5% | 39 | 5.5% | 16 | 9.6% | 7 | 4.3% | 11 | 5.9% | 5 | 2.6% | 53 | 7.5% | 24 | 13.7% | 10 | 6.3% | 17 | 8.1% | 2 | 1.2% |
| Always | 22 | 1.6% | 10 | 1.4% | 3 | 1.8% | 4 | 2.5% | 2 | 1.1% | 1 | 0.5% | 12 | 1.7% | 4 | 2.3% | 3 | 1.9% | 3 | 1.4% | 2 | 1.2% |
| **d25 Had to suppress urge because I can only defecate at certain times of the day** | | | | | | | | | | | | | | | | | | | | | | |
| Never | 1127 | 80.0% | 569 | 80.9% | 116 | 69.9% | 122 | 75.3% | 157 | 84.9% | 174 | 91.6% | 558 | 79.1% | 125 | 71.4% | 121 | 76.6% | 164 | 78.1% | 148 | 91.4% |
| Sometimes | 239 | 17.0% | 109 | 15.5% | 44 | 26.5% | 32 | 19.8% | 20 | 10.8% | 13 | 6.8% | 130 | 18.4% | 43 | 24.6% | 32 | 20.3% | 41 | 19.5% | 14 | 8.6% |
| Often | 34 | 2.4% | 21 | 3.0% | 5 | 3.0% | 6 | 3.7% | 7 | 3.8% | 3 | 1.6% | 13 | 1.8% | 6 | 3.4% | 4 | 2.5% | 3 | 1.4% | 0 | 0.0% |
| Always | 8 | 0.6% | 4 | 0.6% | 1 | 0.6% | 2 | 1.2% | 1 | 0.5% | 0 | 0.0% | 4 | 0.6% | 1 | 0.6% | 1 | 0.6% | 2 | 1.0% | 0 | 0.0% |
| **d26 Feared I would be harmed by someone when I went to defecate^2^** | | | | | | | | | | | | | | | | | | | | | | |
| Never | 1336 | 94.9% | 678 | 96.4% | 161 | 97.0% | 159 | 98.1% | 176 | 95.1% | 182 | 95.8% | 658 | 93.3% | 154 | 88.0% | 150 | 94.9% | 199 | 94.8% | 155 | 95.7% |
| Sometimes | 38 | 2.7% | 12 | 1.7% | 2 | 1.2% | 2 | 1.2% | 4 | 2.2% | 4 | 2.1% | 26 | 3.7% | 10 | 5.7% | 3 | 1.9% | 8 | 3.8% | 5 | 3.1% |
| Often | 8 | 0.6% | 4 | 0.6% | 0 | 0.0% | 0 | 0.0% | 4 | 2.2% | 0 | 0.0% | 4 | 0.6% | 2 | 1.1% | 1 | 0.6% | 0 | 0.0% | 1 | 0.6% |
| Always | 26 | 1.8% | 9 | 1.3% | 3 | 1.8% | 1 | 0.6% | 1 | 0.5% | 4 | 2.1% | 17 | 2.4% | 9 | 5.1% | 4 | 2.5% | 3 | 1.4% | 1 | 0.6% |
| **d27 Had trouble finding someone to watch dependents (like children, sick or elderly) so I could defecate^2^** | | | | | | | | | | | | | | | | | | | | | | |
| Never | 1253 | 89.0% | 640 | 91.0% | 163 | 98.2% | 114 | 70.4% | 175 | 94.6% | 188 | 98.9% | 613 | 87.0% | 172 | 98.3% | 109 | 69.0% | 175 | 83.3% | 157 | 96.9% |
| Sometimes | 110 | 7.8% | 46 | 6.5% | 2 | 1.2% | 36 | 22.2% | 6 | 3.2% | 2 | 1.1% | 64 | 9.1% | 2 | 1.1% | 34 | 21.5% | 24 | 11.4% | 4 | 2.5% |
| Often | 36 | 2.6% | 12 | 1.7% | 0 | 0.0% | 10 | 6.2% | 2 | 1.1% | 0 | 0.0% | 24 | 3.4% | 0 | 0.0% | 12 | 7.6% | 11 | 5.2% | 1 | 0.6% |
| Always | 9 | 0.6% | 5 | 0.7% | 1 | 0.6% | 2 | 1.2% | 2 | 1.1% | 0 | 0.0% | 4 | 0.6% | 1 | 0.6% | 3 | 1.9% | 0 | 0.0% | 0 | 0.0% |
| **Supplementary Table S2d: Frequency of participant responses for defecation module questions by random split halves and life stage categories. *(Continued).***  ***Question:*** ***How often have you experienced any of the following in the previous 30 days when going to defecate?*** | | | | | | | | | | | | | | | | | | | | | | |
|  | ***Full Sample*** | | ***Sub-Sample N_1_*** | | | | | | | | | | ***Sub-Sample N_2_*** | | | | | | | | | |
|  | All  N=1408 | | All  N_1_=703 | | 1. Unmarried (UM)  n=166 | | 2. Recently Married (RM) n=162 | | 3. Married (M)  n=185 | | 4. Over 49 (OW)  n=190 | | All  N_2_=705 | | 1. Unmarried (UM)  n=175 | | 2. Recently Married (RM) n=158 | | 3. Married (M)  n=210 | | 4. Over 49 (OW)  n=162 | |
|  |  |  |  |  |  |  |  |  |  |  |  |  |  |  |  |  |  |  |  |  |  |  |
| **d28 Had trouble controlling urge to defecate** | | | | | | | | | | | | | | | | | | | | | | |
| Never | 1107 | 78.2% | 558 | 79.4% | 128 | 77.1% | 125 | 77.2% | 143 | 77.3% | 162 | 85.3% | 549 | 77.9% | 131 | 74.9% | 124 | 78.5% | 164 | 78.1% | 130 | 80.2% |
| Sometimes | 254 | 17.9% | 124 | 17.6% | 35 | 21.1% | 32 | 19.8% | 35 | 18.9% | 22 | 11.6% | 130 | 18.4% | 36 | 20.6% | 27 | 17.1% | 42 | 20.0% | 25 | 15.4% |
| Often | 31 | 2.2% | 16 | 2.3% | 3 | 1.8% | 2 | 1.2% | 6 | 3.2% | 5 | 2.6% | 15 | 2.1% | 6 | 3.4% | 4 | 2.5% | 3 | 1.4% | 2 | 1.2% |
| Always | 16 | 1.1% | 5 | 0.7% | 0 | 0.0% | 3 | 1.9% | 1 | 0.5% | 1 | 0.5% | 11 | 1.6% | 2 | 1.1% | 3 | 1.9% | 1 | 0.5% | 5 | 3.1% |
| **d29 Worried about defecating in the same place as others** | | | | | | | | | | | | | | | | | | | | | | |
| Never | 858 | 60.9% | 428 | 60.9% | 79 | 47.6% | 107 | 66.0% | 108 | 58.4% | 134 | 70.5% | 430 | 61.0% | 84 | 48.0% | 104 | 65.8% | 123 | 58.6% | 119 | 73.5% |
| Sometimes | 199 | 14.1% | 101 | 14.4% | 36 | 21.7% | 14 | 8.6% | 27 | 14.6% | 24 | 12.6% | 98 | 13.9% | 33 | 18.9% | 17 | 10.8% | 32 | 15.2% | 16 | 9.9% |
| Often | 101 | 7.2% | 48 | 6.8% | 14 | 8.4% | 7 | 4.3% | 17 | 9.2% | 10 | 5.3% | 53 | 7.5% | 13 | 7.4% | 8 | 5.1% | 20 | 9.5% | 12 | 7.4% |
| Always | 150 | 10.7% | 126 | 17.9% | 37 | 22.3% | 34 | 21.0% | 33 | 17.8% | 22 | 11.6% | 124 | 17.6% | 45 | 25.7% | 29 | 18.4% | 35 | 16.7% | 16 | 9.9% |
| **d30 Had to stand while defecating because someone came** | | | | | | | | | | | | | | | | | | | | | | |
| Never | 627 | 44.5% | 310 | 44.1% | 49 | 29.5% | 87 | 53.7% | 66 | 35.7% | 108 | 56.8% | 317 | 45.0% | 59 | 33.7% | 85 | 53.8% | 80 | 38.1% | 93 | 57.4% |
| Sometimes | 614 | 43.6% | 310 | 44.1% | 89 | 53.6% | 60 | 37.0% | 92 | 49.7% | 69 | 36.3% | 304 | 43.1% | 79 | 45.1% | 60 | 38.0% | 102 | 48.6% | 63 | 38.9% |
| Often | 130 | 9.2% | 63 | 9.0% | 19 | 11.4% | 10 | 6.2% | 22 | 11.9% | 12 | 6.3% | 67 | 9.5% | 29 | 16.6% | 12 | 7.6% | 21 | 10.0% | 5 | 3.1% |
| Always | 37 | 2.6% | 20 | 2.8% | 9 | 5.4% | 5 | 3.1% | 5 | 2.7% | 1 | 0.5% | 17 | 2.4% | 8 | 4.6% | 1 | 0.6% | 7 | 3.3% | 1 | 0.6% |
| **d31 Withheld food to control urge to defecate^1^** | | | | | | | | | | | | | | | | | | | | | | |
| Never | 1296 | 92.1% | 648 | 92.2% | 153 | 92.2% | 149 | 92.0% | 171 | 92.4% | 175 | 92.6% | 648 | 92.0% | 160 | 91.4% | 142 | 89.9% | 196 | 93.3% | 150 | 92.6% |
| Sometimes | 83 | 5.9% | 44 | 6.3% | 12 | 7.2% | 9 | 5.6% | 11 | 5.9% | 12 | 6.3% | 39 | 5.5% | 9 | 5.1% | 10 | 6.3% | 12 | 5.7% | 8 | 4.9% |
| Often | 24 | 1.7% | 9 | 1.3% | 1 | 0.6% | 3 | 1.9% | 3 | 1.6% | 2 | 1.1% | 15 | 2.1% | 4 | 2.3% | 5 | 3.2% | 2 | 1.0% | 4 | 2.5% |
| Always | 4 | 0.3% | 1 | 0.1% | 0 | 0.0% | 1 | 0.6% | 0 | 0.0% | 0 | 0.0% | 4 | 0.6% | 2 | 1.1% | 1 | 0.6% | 0 | 0.0% | 0 | 0.0% |
| **d32 Worried others would get upset if asked to accompany for defecation** | | | | | | | | | | | | | | | | | | | | | | |
| Never | 1260 | 89.5% | 629 | 89.5% | 140 | 84.3% | 140 | 86.4% | 169 | 91.4% | 180 | 94.7% | 631 | 89.5% | 151 | 86.3% | 137 | 86.7% | 191 | 91.0% | 152 | 93.8% |
| Sometimes | 115 | 8.2% | 60 | 8.5% | 20 | 12.0% | 17 | 10.5% | 14 | 7.6% | 9 | 4.7% | 55 | 7.8% | 18 | 10.3% | 14 | 8.9% | 18 | 8.6% | 5 | 3.1% |
| Often | 16 | 1.1% | 8 | 1.1% | 3 | 1.8% | 3 | 1.9% | 1 | 0.5% | 1 | 0.5% | 8 | 1.1% | 2 | 1.1% | 4 | 2.5% | 1 | 0.5% | 1 | 0.6% |
| Always | 17 | 1.2% | 6 | 0.9% | 3 | 1.8% | 2 | 1.2% | 1 | 0.5% | 0 | 0.0% | 11 | 1.6% | 4 | 2.3% | 3 | 1.9% | 0 | 0.0% | 4 | 2.5% |
| **d33 Worried about dependents (children, sick or elderly) who need me when I go to defecate^2^** | | | | | | | | | | | | | | | | | | | | | | |
| Never | 1248 | 88.6% | 640 | 91.0% | 162 | 97.6% | 119 | 73.5% | 171 | 92.4% | 188 | 98.9% | 608 | 86.2% | 169 | 96.6% | 106 | 67.1% | 179 | 85.2% | 154 | 95.1% |
| Sometimes | 80 | 5.7% | 28 | 4.0% | 3 | 1.8% | 16 | 9.9% | 7 | 3.8% | 2 | 1.1% | 52 | 7.4% | 2 | 1.1% | 28 | 17.7% | 16 | 7.6% | 6 | 3.7% |
| Often | 44 | 3.1% | 18 | 2.6% | 1 | 0.6% | 13 | 8.0% | 4 | 2.2% | 0 | 0.0% | 26 | 3.7% | 1 | 0.6% | 16 | 10.1% | 9 | 4.3% | 0 | 0.0% |
| Always | 36 | 2.6% | 17 | 2.4% | 0 | 0.0% | 14 | 8.6% | 3 | 1.6% | 0 | 0.0% | 19 | 2.7% | 3 | 1.7% | 8 | 5.1% | 6 | 2.9% | 2 | 1.2% |
| **d34 Worried that I have no money to build or maintain a toilet** | | | | | | | | | | | | | | | | | | | | | | |
| Never | 563 | 40.0% | 275 | 39.1% | 52 | 31.3% | 83 | 51.2% | 61 | 33.0% | 79 | 41.6% | 288 | 40.9% | 64 | 36.6% | 82 | 51.9% | 73 | 34.8% | 69 | 42.6% |
| Sometimes | 110 | 7.8% | 55 | 7.8% | 13 | 7.8% | 8 | 4.9% | 15 | 8.1% | 19 | 10.0% | 55 | 7.8% | 17 | 9.7% | 11 | 7.0% | 9 | 4.3% | 18 | 11.1% |
| Often | 98 | 7.0% | 51 | 7.3% | 12 | 7.2% | 5 | 3.1% | 21 | 11.4% | 13 | 6.8% | 47 | 6.7% | 9 | 5.1% | 6 | 3.8% | 20 | 9.5% | 12 | 7.4% |
| Always | 637 | 45.2% | 322 | 45.8% | 89 | 53.6% | 66 | 40.7% | 88 | 47.6% | 79 | 41.6% | 315 | 44.7% | 85 | 48.6% | 59 | 37.3% | 108 | 51.4% | 63 | 38.9% |
| **d35 Had men or boys harm or harass me when going to defecate** | | | | | | | | | | | | | | | | | | | | | | |
| Never | 1406 | 99.9% | 702 | 99.9% | 165 | 99.4% | 162 | 100% | 185 | 100% | 190 | 100% | 704 | 99.9% | 174 | 99.4% | 158 | 100% | 210 | 100% | 162 | 100% |
| Sometimes | 1 | 0.1% | 1 | 0.1% | 1 | 0.6% | 0 | 0.0% | 0 | 0.0% | 0 | 0.0% | 0 | 0.0% | 0 | 0.0% | 0 | 0.0% | 0 | 0.0% | 0 | 0.0% |
| Often | 0 | 0.0% | 0 | 0.0% | 0 | 0.0% | 0 | 0.0% | 0 | 0.0% | 0 | 0.0% | 0 | 0.0% | 0 | 0.0% | 0 | 0.0% | 0 | 0.0% | 0 | 0.0% |
| Always | 1 | 0.1% | 0 | 0.0% | 0 | 0.0% | 0 | 0.0% | 0 | 0.0% | 0 | 0.0% | 1 | 0.1% | 1 | 0.6% | 0 | 0.0% | 0 | 0.0% | 0 | 0.0% |

| **Supplementary Table S2e: Frequency of participant responses for defecation module questions by random split halves and life stage categories. *(Continued).***  ***Question:*** ***How often have you experienced any of the following in the previous 30 days when going to defecate?*** | | | | | | | | | | | | | | | | | | | | | | |
| --- | --- | --- | --- | --- | --- | --- | --- | --- | --- | --- | --- | --- | --- | --- | --- | --- | --- | --- | --- | --- | --- | --- |
|  | ***Full Sample*** | | ***Sub-Sample N_1_*** | | | | | | | | | | ***Sub-Sample N_2_*** | | | | | | | | | |
|  | All  N=1408 | | All  N_1_=703 | | 1. Unmarried (UM)  n=166 | | 2. Recently Married (RM) n=162 | | 3. Married (M)  n=185 | | 4. Over 49 (OW)  n=190 | | All  N_2_=705 | | 1. Unmarried (UM)  n=175 | | 2. Recently Married (RM) n=158 | | 3. Married (M)  n=210 | | 4. Over 49 (OW)  n=162 | |
|  |  |  |  |  |  |  |  |  |  |  |  |  |  |  |  |  |  |  |  |  |  |  |
| **d36 Have had to go back and forth to defecation location because could not find privacy** | | | | | | | | | | | | | | | | | | | | | | |
| Never | 928 | 65.9% | 474 | 67.4% | 93 | 56.0% | 118 | 72.8% | 125 | 67.6% | 138 | 72.6% | 454 | 64.4% | 102 | 58.3% | 115 | 72.8% | 128 | 61.0% | 109 | 67.3% |
| Sometimes | 326 | 23.2% | 155 | 22.0% | 50 | 30.1% | 23 | 14.2% | 39 | 21.1% | 43 | 22.6% | 171 | 24.3% | 50 | 28.6% | 28 | 17.7% | 54 | 25.7% | 39 | 24.1% |
| Often | 131 | 9.3% | 66 | 9.4% | 19 | 11.4% | 20 | 12.3% | 19 | 10.3% | 8 | 4.2% | 65 | 9.2% | 14 | 8.0% | 13 | 8.2% | 25 | 11.9% | 13 | 8.0% |
| Always | 23 | 1.6% | 8 | 1.1% | 4 | 2.4% | 1 | 0.6% | 2 | 1.1% | 1 | 0.5% | 15 | 2.1% | 9 | 5.1% | 2 | 1.3% | 3 | 1.4% | 1 | 0.6% |
| 1. Missings: D01, 1 missing (N_1_, stage D); D31, 1 missing (N1, stage D)  2. Chi-Square significant differences: D11, Stage A p=0.0.0236; D14, by overall split half group p=0.0069 and Stage A, C=0.0004; D18, Stage A p=0.0287; D21, Stage A p=0.0284; D22, Stage D p=0.0410; D23, Stage D p=0.0434; D26, Stages A: p=0.0165; D27, Stage C p=0.0006; D33, by overall split half group p=0.0225 | | | | | | | | | | | | | | | | | | | | | | |

| **Supplementary Table S3a: Factor loadings, factor co-variations, and model fit statistics for random split-half sample EFA (N_1_=703) and CFA models (N_2_=705), baseline and final MIMIC models (N_2_=708), and final CFA model (N_2_=708) with deletions based on DIF*.*** | | | | | | | | | | | | |
| --- | --- | --- | --- | --- | --- | --- | --- | --- | --- | --- | --- | --- |
|  |  |  |  |  |  |  |  |  |  |  |  |  |
|  | **Item** | **EFA (N_1_=703)** | | **CFA (N_2_=705)** | | **Baseline MIMIC Model (N_2_=705)** | | **Final MIMIC Model, 10 Modifications (N_2_=705)** | | **CFA, with deletions based on DIF (N_2_=705)** | |  |
|  |  |  |  |  |  |  |  |  |  |  |  |  |
| **Factors** |  |  |  |  |  |  |  |  |  |  |  |  |
| ***Factor 1: Potential Harms*** |  |  |  |  |  |  |  |  |  |  |  |  |
| Worried about not having a proper facility to urinate | U01 | 0.697 |  | 0.825 | * | 0.830 | * | 0.830 | * | 0.824 | * |  |
| Worried that someone would see me while urinating | U03 | 0.714 |  | 0.828 | * | 0.824 | * | 0.824 | * | 0.819 | * |  |
| Had difficulty finding clean place to urinate | U06 | 0.773 |  | 0.883 | * | 0.889 | * | 0.889 | * | 0.885 | * |  |
| Felt worried that I would step on urine | U08 | 0.758 |  | 0.853 | * | 0.856 | * | 0.856 | * | 0.855 | * |  |
| Felt concerned I would get an infection if I was urinating in an unsuitable/dirty place | U10 | 0.822 |  | 0.963 | * | 0.962 | * | 0.962 | * | 0.964 | * |  |
| Feared I would be harmed by animals or insects when I went to urinate | U11 | 0.821 |  | 0.806 | * | 0.799 | * | 0.799 | * | 0.811 | * |  |
| Feared I would be harmed by someone when I went to urinate | U12 | 0.910 |  | 0.817 | * | 0.803 | * | 0.803 | * | 0.825 | * |  |
| Felt concerned I would get an infection if I urinated on someone else’s urine | U15 | 0.810 |  | 0.953 | * | 0.951 | * | 0.951 | * | 0.956 | * |  |
| Feared I would be harmed by animals or insects when I went to defecate | D12 | 0.791 |  | 0.724 | * | 0.733 | * | 0.733 | * | 0.717 | * |  |
| Worried about getting an infection when going to defecate | D15 | 0.779 |  | 0.958 | * | 0.961 | * | 0.961 | * | 0.943 | * |  |
| Feared I would be harmed by someone when I went to defecate | D26 | 0.706 |  | 0.798 | * | 0.808 | * | 0.808 | * | 0.794 | * |  |
| **Factor 2:  *Social expectations resultant repercussions*** |  |  |  |  |  |  |  |  |  |  |  |  |
| Could not always go to urinate when there was a need | U02 | 0.766 |  | 0.680 | * | 0.695 | * | 0.694 | * | 0.681 | * |  |
| Experienced difficulty controlling urge to urinate | U04 | 0.702 |  | 0.658 | * | 0.681 | * | 0.680 | * | 0.656 | * |  |
| Worried people would talk about me if they saw me | U09 | 0.863 |  | 0.785 | * | 0.780 | * | 0.779 | * | 0.792 | * |  |
| Had difficulty finding a private place to urinate | U17 | 0.760 |  | 0.866 | * | 0.864 | * | 0.863 | * | 0.871 | * |  |
| Had to suppress urge because people were around and could not go | U20 | 0.766 |  | 0.852 | * | 0.850 | * | 0.849 | * | 0.855 | * |  |
| Had to stand while urinating because someone came | U25 | 0.610 |  | 0.760 | * | 0.762 | * | 0.761 | * | 0.752 | * |  |
| Had to suppress urge because did not have someone to accompany me^✢^ | U26 | 0.773 |  | 0.724 | * | 0.723 | * | 0.676 | * | - |  |  |
| Had to suppress [urination] when workload was high | U29 | 0.594 |  | 0.587 | * | 0.583 | * | 0.582 | * | 0.580 | * |  |
| Worried others would get upset if asked to accompany for urination | U31 | 0.856 |  | 0.707 | * | 0.704 | * | 0.703 | * | 0.684 | * |  |
| Had to suppress when I got an urge at night | U32 | 0.704 |  | 0.593 | * | 0.598 | * | 0.597 | * | 0.586 | * |  |
| Had to suppress urge [to defecate] when workload was high | D09 | 0.563 |  | 0.656 | * | 0.655 | * | 0.654 | * | 0.636 | * |  |
| Worried people would talk about me if they saw me | D11 | 0.803 |  | 0.816 | * | 0.814 | * | 0.814 | * | 0.811 | * |  |
| Had to suppress urge because I can only defecate at certain times of the day | D25 | 0.661 |  | 0.792 | * | 0.790 | * | 0.789 | * | 0.771 | * |  |
| Had trouble controlling urge to defecate | D28 | 0.553 |  | 0.700 | * | 0.729 | * | 0.728 | * | 0.695 | * |  |
| **Factor 3:  *Physical exertion or strain*** |  |  |  |  |  |  |  |  |  |  |  |  |
| Experienced pain during urination^✢^ | U05 | 0.601 |  | 0.583 | * | 0.639 | * | 0.636 | * | - |  |  |
| Had difficulty accessing water for urination | U19 | 0.612 |  | 0.833 | * | 0.872 | * | 0.873 | * | 0.852 | * |  |
| Had frequent pressure to urinate^✢^ | U22 | 0.431 |  | - |  | - |  | - |  | - |  |  |
| Had to do extra work washing clothes because of dirty conditions where urinating | U23 | 0.715 |  | 0.758 | * | 0.755 | * | 0.758 | * | 0.752 | * |  |
| Withheld water to control urge to urinate | U28 | 0.387 |  | 0.567 | * | 0.568 | * | 0.571 | * | 0.564 | * |  |
| Experienced pain during defecation^✢^ | D04 | 0.492 |  | 0.370 | * | 0.433 | * | 0.426 | * | - |  |  |
| Had difficulty accessing water for defecation | D21 | 0.674 |  | 0.906 | * | 0.882 | * | 0.884 | * | 0.915 | * |  |
| Had difficulty cleaning/washing myself after defecation | D23 | 0.709 |  | 0.835 | * | 0.825 | * | 0.828 | * | 0.848 | * |  |
| Withheld food to control urge to defecate | D31 | 0.585 |  | 0.694 | * | 0.700 | * | 0.703 | * | 0.669 | * |  |
|  |  |  |  |  |  |  |  |  |  |  |  |  |
| **Supplementary Table S3b: Factor loadings, factor co-variations, and model fit statistics for random split-half sample EFA (N_1_=703) and CFA models (N_2_=705), baseline and final MIMIC models (N_2_=708), and final CFA model (N_2_=708) with deletions based on DIF*. (Continued).*** | | | | | | | | | | | | |
|  |  |  |  |  |  |  |  |  |  |  |  |  |
|  | **Item** | **EFA (N_1_=703)** | | **CFA (N_2_=705)** | | **Baseline MIMIC Model (N_2_=705)** | | **Final MIMIC Model, 10 Modifications (N_2_=705)** | | **CFA, with deletions based on DIF (N_2_=705)** | |  |
|  |  |  |  |  |  |  |  |  |  |  |  |  |
| **Factors** |  |  |  |  |  |  |  |  |  |  |  |  |
| **Factor 4: *Night Concerns*** |  |  |  |  |  |  |  |  |  |  |  |  |
| Felt scared urinating in the dark at night | U13 | 0.809 |  | 0.919 | * | 0.923 | * | 0.923 | * | 0.920 | * |  |
| Felt scared of ghosts when I went to urinate at night | U16 | 0.870 |  | 0.950 | * | 0.946 | * | 0.946 | * | 0.952 | * |  |
| Felt scared defecating in the dark at night | D10 | 0.722 |  | 0.918 | * | 0.920 | * | 0.920 | * | 0.914 | * |  |
| Felt scared of ghosts when I went to defecate at night | D18 | 0.793 |  | 0.915 | * | 0.918 | * | 0.918 | * | 0.914 | * |  |
| **Factor 5: S*ocial support*** |  |  |  |  |  |  |  |  |  |  |  |  |
| Had to leave dependents (like children, sick, or elderly) alone to urinate | U24 | 0.907 |  | 0.889 | * | 0.880 | * | 0.897 | * | 0.915 | * |  |
| Had trouble finding someone to watch dependents (children, sick, elderly) so I could urinate | U27 | 0.928 |  | 0.939 | * | 0.945 | * | 0.956 | * | 0.962 | * |  |
| Had to find someone to look after my work so I could defecate^✢^ | D20 | 0.619 |  | 0.791 | * | 0.769 | * | 0.881 | * | - |  |  |
| Had trouble finding someone to watch dependents so I could defecate | D27 | 0.933 |  | 0.919 | * | 0.913 | * | 0.928 | * | 0.942 | * |  |
| Worried others would get upset if asked to accompany for defecation^✢^ | D32 | 0.481 |  | 0.876 | * | 0.867 | * | 1.033 | * | - |  |  |
| Worried about dependents (children, sick or elderly) who need me when I go to defecate | D33 | 0.920 |  | 0.906 | * | 0.905 | * | 0.918 | * | 0.933 | * |  |
| **Factor 6: *Physical agility*** |  |  |  |  |  |  |  |  |  |  |  |  |
| Had difficulty or pain sitting or getting up for urination | U18 | 0.878 |  | 0.925 | * | 0.920 | * | 0.920 | * | 0.920 | * |  |
| Worried I would fall when going to defecate | D07 | 0.801 |  | 0.758 | * | 0.782 | * | 0.782 | * | 0.763 | * |  |
| Had difficulty or pain squatting for defecation | D17 | 0.920 |  | 0.951 | * | 0.936 | * | 0.936 | * | 0.954 | * |  |
| Had difficulty walking to defecation place^✢^ | D19 | 0.713 |  | - |  | - |  | - |  | - |  |  |
|  |  |  |  |  |  |  |  |  |  |  |  |  |
| **Factor 7:  *Defecation place*** |  |  |  |  |  |  |  |  |  |  |  |  |
| Worried about not having a toilet to defecate | D01 | 0.945 |  | 0.885 | * | 0.886 | * | 0.885 | * | 0.900 | * |  |
| Had to go far to defecate | D02 | 0.851 |  | 0.799 | * | 0.806 | * | 0.804 | * | 0.808 | * |  |
| Defecation process/ activity of defecation took a long time to complete | D03 | 0.804 |  | 0.782 | * | 0.797 | * | 0.795 | * | 0.801 | * |  |
| Had difficulty finding a clean place to defecate | D05 | 0.879 |  | 0.876 | * | 0.884 | * | 0.884 | * | 0.888 | * |  |
| Could not access preferred location | D06 | 0.739 |  | 0.865 | * | 0.870 | * | 0.869 | * | 0.885 | * |  |
| Worried that someone would see me defecating^✢^ | D08 | 0.828 |  | 0.869 | * | 0.859 | * | 0.813 | * | - |  |  |
| Had to do extra work washing clothes because of dirty conditions where defecating | D14 | 0.683 |  | 0.770 | * | 0.772 | * | 0.770 | * | 0.779 | * |  |
| Had to suppress the urge to defecate because people were around^✢^ | D24 | 0.799 |  | 0.851 | * | 0.845 | * | 0.821 | * | - |  |  |
| Worried about defecating in the same place as others | D29 | 0.852 |  | 0.944 | * | 0.938 | * | 0.937 | * | 0.963 | * |  |
| Worried have no money to build or maintain toilet | D34 | 0.913 |  | 0.872 | * | 0.875 | * | 0.874 | * | 0.890 | * |  |
| Have had to go back and forth to defecation location because could not find privacy | D35 | 0765 |  | 0.860 | * | 0.874 | * | 0.873 | * | 0.860 | * |  |

| **Supplementary Table S3c: Factor loadings, factor co-variations, and model fit statistics for random split-half sample EFA (N_1_=703) and CFA models (N_2_=705), baseline and final MIMIC models (N_2_=708), and final CFA model (N_2_=708) with deletions based on DIF*. (Continued).*** | | | | | | | | | | | | |
| --- | --- | --- | --- | --- | --- | --- | --- | --- | --- | --- | --- | --- |
|  |  |  |  |  |  |  |  |  |  |  |  |  |
|  | **Item** | **EFA (N_1_=703)** | | **CFA (N_2_=705)** | | **Baseline MIMIC Model (N_2_=705)** | | **Final MIMIC Model, 10 Modifications (N_2_=705)** | | **CFA, with deletions based on DIF (N_2_=705)** | |  |
|  |  |  |  |  |  |  |  |  |  |  |  |  |
| **Factors** |  |  |  |  |  |  |  |  |  |  |  |  |
| **Factor Covariates** |  |  |  |  |  |  |  |  |  |  |  | |
| Factor 2 |  |  |  |  |  |  |  |  |  |  |  | |
| *With Factor 1* |  |  |  | 0.770 | * | 0.756 | * | 0.756 | * | 0.780 | * | |
| Factor 3 |  |  |  |  |  |  |  |  |  |  |  | |
| *With Factor 1* |  |  |  | 0.488 | * | 0.486 | * | 0.486 | * | 0.516 |  | |
| *With Factor 2* |  |  |  | 0.583 | * | 0.585 | * | 0.585 | * | 0.570 | * | |
| Factor 4 |  |  |  |  |  |  |  |  |  |  |  | |
| *With Factor 1* |  |  |  | 0.697 | * | 0.675 | * | 0.675 | * | 0.698 | * | |
| *With Factor 2* |  |  |  | 0.601 | * | 0.578 | * | 0.578 | * | 0.604 | * | |
| *With Factor 3* |  |  |  | 0.401 | * | 0.398 | * | 0.398 | * | 0.415 | * | |
| Factor 5 |  |  |  |  |  |  |  |  |  |  |  | |
| *With Factor 1* |  |  |  | 0.463 | * | 0.484 | * | 0.483 | * | 0.424 | * | |
| *With Factor 2* |  |  |  | 0.538 | * | 0.549 | * | 0.548 | * | 0.440 | * | |
| *With Factor 3* |  |  |  | 0.406 | * | 0.439 | * | 0.438 | * | 0.352 | * | |
| *With Factor 4* |  |  |  | 0.433 | * | 0.416 | * | 0.416 | * | 0.387 | * | |
| Factor 6 |  |  |  |  |  |  |  |  |  |  |  | |
| *With Factor 1* |  |  |  | 0.243 | * | 0.469 | * | 0.469 | * | 0.244 | * | |
| *With Factor 2* |  |  |  | 0.387 | * | 0.614 | * | 0.614 | * | 0.385 | * | |
| *With Factor 3* |  |  |  | 0.573 | * | 0.671 | * | 0.671 | * | 0.437 | * | |
| *With Factor 4* |  |  |  | 0.069 |  | 0.353 | * | 0.353 | * | 0.068 |  | |
| *With Factor 5* |  |  |  | 0.217 | * | 0.410 | * | 0.409 | * | 0.166 | * | |
| Factor 7 |  |  |  |  |  |  |  |  |  |  |  | |
| *With Factor 1* |  |  |  | 0.782 | * | 0.784 | * | 0.784 | * | 0.770 | * | |
| **Model Fit Statistics** |  |  |  |  |  |  |  |  |  |  |  | |
| RMSEA |  | 0.034 |  | 0.057 |  | 0.055 |  | 0.055 |  | 0.060 |  | |
| CFI^✢✢^ |  | - |  | 0.936 |  | 0.934 |  | 0.935 |  | 0.944 |  | |
| TLI^✢✢^ |  | - |  | 0.933 |  | 0.930 |  | 0.931 |  | 0.941 |  | |
| *p ≤ 0.050. | | | | | | | | | | | | |
| ✢Items in initial EFA model but removed during CFA because of a negative variance (D19) or low factor loading of <0.150 (U22), or later deleted as a result of DIF. | | | | | | | | | | | | |
| ✢✢CFI and TLI not provided in MPLUS for EFA carried out with PROMAX rotation. | | | | | | | | | | | | |
|  |  |  |  |  |  |  |  |  |  |  |  | |

| **Supplementary Table S4a: Structural Regressions, Direct Effects, and Model Fit Statistics of MIMIC Models (N_2_=705)** | | | | | | | | | | | | | | | | | | |  |
| --- | --- | --- | --- | --- | --- | --- | --- | --- | --- | --- | --- | --- | --- | --- | --- | --- | --- | --- | --- |
|  | **Baseline MIMIC Model (N_2_=705)** | **MIMIC Model,**  **1 Mod. (N_2_=705)** | **MIMIC Model,**  **2 Mods. (N_2_=705)** | **MIMIC Model,**  **3 Mods. (N_2_=705)** | **MIMIC Model,**  **4 Mods. (N_2_=705)** | | **MIMIC Model,**  **5 Mods. (N_2_=705)** | | **MIMIC Model,**  **6 Mods. (N_2_=705)** | | **MIMIC Model,**  **7 Mods. (N_2_=705)** | **MIMIC Model,**  **8 Mods. (N_2_=705)** | | **MIMIC Model,**  **9 Mods. (N_2_=705)** | | **Final MIMIC Model,**  **10 Mods. (N_2_=705)** | |  |  |
|  |  |  |  |  |  |  |  |  |  |  |  |  |  |  |  |  |  |  |  |
|  |  |  |  |  |  |  |  |  |  |  |  |  |  |  |  |  |  |  |  |
| **Structural Regressions (Indirect Effects; Ref: Stage 1: Adolescents)** | | | | | | | | | | | | | | | | | | | |
| *On Factor 1* |  |  |  |  | |  | |  | |  |  | |  | |  | |  | | |
| Stage 2: Recently Married Women | -0.064 | -0.064 | -0.064 | -0.064 | | -0.064 | | -0.064 | | -0.064 | -0.064 | | -0.064 | | -0.064 | | -0.064 | | |
| Stage 3: Married Women (> 3 yrs.) | -0.104* | -0.104* | -0.104* | -0.104* | | -0.104* | | -0.104* | | -0.104* | -0.104* | | -0.104* | | -0.104* | | -0.104* | | |
| Stage 4: Women over age 49 | -0.296* | -0.296* | -0.296* | -0.296* | | -0.296* | | -0.296* | | -0.296* | -0.296* | | -0.296* | | -0.296* | | -0.296* | | |
| *On Factor 2* |  |  |  |  | |  | |  | |  |  | |  | |  | |  | | |
| Stage 2: Recently Married Women | -0.001 | -0.001 | -0.001 | -0.001 | | -0.001 | | -0.001 | | -0.001 | -0.001 | | -0.001 | | -0.001 | | -0.001 | | |
| Stage 3: Married Women (> 3 yrs.) | -0.036 | -0.036 | -0.036 | -0.036 | | -0.036 | | -0.036 | | -0.036 | -0.036 | | -0.036 | | -0.036 | | -0.036 | | |
| Stage 4: Women over age 49 | -0.239* | -0.239* | -0.239* | -0.239* | | -0.239* | | -0.239* | | -0.239* | -0.239* | | -0.227* | | -0.227* | | -0.227* | | |
| *On Factor 3* |  |  |  |  | |  | |  | |  |  | |  | |  | |  | | |
| Stage 2: Recently Married Women | 0.055 | 0.055 | 0.055 | 0.055 | | 0.055 | | 0.055 | | 0.055 | 0.055 | | 0.055 | | 0.055 | | 0.055 | | |
| Stage 3: Married Women (> 3 yrs.) | -0.041 | -0.041 | -0.041 | -0.041 | | -0.041 | | -0.041 | | -0.041 | -0.041 | | -0.041 | | -0.041 | | -0.041 | | |
| Stage 4: Women over age 49 | -0.023 | -0.023 | -0.023 | -0.023 | | -0.076 | | -0.076 | | -0.140* | -0.140* | | -0.140* | | -0.140* | | -0.140* | | |
| *On Factor 4* |  |  |  |  | |  | |  | |  |  | |  | |  | |  | | |
| Stage 2: Recently Married Women | -0.028 | -0.028 | -0.028 | -0.028 | | -0.028 | | -0.028 | | -0.028 | -0.028 | | -0.028 | | -0.028 | | -0.028 | | |
| Stage 3: Married Women (> 3 yrs.) | -0.226* | -0.226* | -0.226* | -0.226* | | -0.226* | | -0.226* | | -0.226* | -0.226* | | -0.226* | | -0.226* | | -0.226* | | |
| Stage 4: Women over age 49 | -0.425* | -0.425* | -0.425* | -0.425* | | -0.425* | | -0.425* | | -0.425* | -0.425* | | -0.425* | | -0.425* | | -0.425* | | |
| *On Factor 5* |  |  |  |  | |  | |  | |  |  | |  | |  | |  | | |
| Stage 2: Recently Married Women | 0.350* | 0.431* | 0.427* | 0.521* | | 0.521* | | 0.521* | | 0.521* | 0.513* | | 0.513* | | 0.513* | | 0.513* | | |
| Stage 3: Married Women (> 3 yrs.) | 0.208* | 0.201* | 0.278* | 0.264* | | 0.264* | | 0.264* | | 0.264* | 0.354* | | 0.354* | | 0.354* | | 0.354* | | |
| Stage 4: Women over age 49 | -0.091 | -0.088 | -0.088 | -0.084 | | -0.084 | | -0.084 | | -0.084 | -0.082 | | -0.082 | | -0.082 | | -0.082 | | |
| *On Factor 6* |  |  |  |  | |  | |  | |  |  | |  | |  | |  | | |
| Stage 2: Recently Married Women | 0.110* | 0.110* | 0.110* | 0.110* | | 0.110* | | 0.110* | | 0.110* | 0.110* | | 0.110* | | 0.110* | | 0.110* | | |
| Stage 3: Married Women (> 3 yrs.) | 0.184* | 0.184* | 0.184* | 0.184* | | 0.184* | | 0.184* | | 0.184* | 0.184* | | 0.184* | | 0.184* | | 0.184* | | |
| Stage 4: Women over age 49 | 0.547* | 0.547* | 0.547* | 0.547* | | 0.547* | | 0.547* | | 0.547* | 0.547* | | 0.547* | | 0.547* | | 0.547* | | |
| *On Factor 7* |  |  |  |  | |  | |  | |  |  | |  | |  | |  | | |
| Stage 2: Recently Married Women | -0.178* | -0.178* | -0.178* | -0.178* | | -0.178* | | -0.179* | | -0.179* | -0.179* | | -0.179* | | -0.179* | | -0.179* | | |
| Stage 3: Married Women (> 3 yrs.) | -0.067 | -0.067 | -0.067 | -0.067 | | -0.067 | | -0.067 | | -0.067 | -0.067 | | -0.067 | | -0.068 | | -0.068 | | |
| Stage 4: Women over age 49 | -0.203* | -0.203* | -0.203* | -0.203* | | -0.203* | | -0.184* | | -0.184* | -0.184* | | -0.184* | | -0.167* | | -0.147* | | |

| **Table S4b: Structural Regressions, Direct Effects, and Model Fit Statistics of MIMIC Models (N_2_=705) *(Continued)*** | | | | | | | | | | | | | | | | | | |  |
| --- | --- | --- | --- | --- | --- | --- | --- | --- | --- | --- | --- | --- | --- | --- | --- | --- | --- | --- | --- |
|  | **Baseline MIMIC Model (N_2_=705)** | **MIMIC Model,**  **1 Mod. (N_2_=705)** | **MIMIC Model,**  **2 Mods. (N_2_=705)** | **MIMIC Model,**  **3 Mods. (N_2_=705)** | **MIMIC Model,**  **4 Mods. (N_2_=705)** | | **MIMIC Model,**  **5 Mods. (N_2_=705)** | | **MIMIC Model,**  **6 Mods. (N_2_=705)** | | **MIMIC Model,**  **7 Mods. (N_2_=705)** | **MIMIC Model,**  **8 Mods. (N_2_=705)** | | **MIMIC Model,**  **9 Mods. (N_2_=705)** | | **Final MIMIC Model,**  **10 Mods. (N_2_=705)** | |  |  |
|  |  |  |  |  |  |  |  |  |  |  |  |  |  |  |  |  |  |  |  |
|  |  |  |  |  |  |  |  |  |  |  |  |  |  |  |  |  |  |  |  |
| **Direct Effects (DIF)** |  |  |  |  | |  | |  | |  |  | |  | |  | |  | | |
| **D32** 'Worried others would get upset if asked to  accompany for defecation' (factor 5)  *On S2: Recently Married Women* | | -0.407* | -0.418* | -0.533* | | -0.533* | | -0.533* | | -0.533* | -0.534* | | -0.534* | | -0.534* | | -0.534* | | |
| **D32** 'Worried others would get upset if asked to  accompany for defecation' (factor 5)  *On S3: Married Women (> 3 yrs.)* | |  | -0.399* | -0.397* | | -0.397* | | -0.397* | | -0.397* | -0.494* | | -0.494* | | -0.494* | | -0.494* | | |
| **D20** 'Had to find someone to look after my work so I could  defecate' (factor 5)  *On S2: Recently Married Women* | |  |  | -0.276* | | -0.276* | | -0.276* | | -0.276* | -0.283* | | -0.283* | | -0.283* | | -0.283* | | |
| **D04** 'Experienced pain during defecation' (factor 3)  *On* S4: Women over age 49 | |  |  |  | | 0.266* | | 0.266* | | 0.293* | 0.293* | | 0.293* | | 0.293* | | 0.293* | | |
| **D08** 'Worried that someone would see me defecating'  (factor 7)  *On S4: Women over age 49* | |  |  |  | |  | | -0.233* | | -0.233* | -0.233* | | -0.233* | | -0.247* | | -0.263* | | |
| **U05** 'Experienced pain during urination' (factor 3)  *On S4: Women over age 49* | |  |  |  | |  | |  | | 0.262* | 0.262* | | 0.262* | | 0.262* | | 0.262* | | |
| **D20** 'Had to find someone to look after my work so I could  defecate' (factor 5)  *On S3: Married Women (> 3 yrs.)* | |  |  |  | |  | |  | |  | -0.214* | | -0.214* | | -0.214* | | -0.214* | | |
| **U26** 'Had to suppress urge because did not have  someone to accompany me' (factor 2)  *On S4: Women over age 49* | |  |  |  | |  | |  | |  |  | | -0.230* | | -0.230* | | -0.230* | | |
| **D30** 'Had to stand while defecating because someone  came' (factor 7)  *On S4: Women over age 49* | |  |  |  | |  | |  | |  |  | |  | | -0.154* | | -0.170* | | |
| **D24** 'Had to suppress the urge to defecate because  people were around' (factor 7)  *On S4: Women over age 49* | |  |  |  | |  | |  | |  |  | |  | |  | | -0.174* | | |
| **Model Fit Statistics** |  |  |  |  | |  | |  | |  |  | |  | |  | |  | | |
| RMSEA | 0.055 | 0.055 | 0.055 | 0.055 | | 0.055 | | 0.055 | | 0.055 | 0.055 | | 0.055 | | 0.055 | | 0.055 | | |
| CFI^✢✢^ | 0.934 | 0.934 | 0.934 | 0.934 | | 0.935 | | 0.935 | | 0.935 | 0.935 | | 0.935 | | 0.935 | | 0.935 | | |
| TLI^✢✢^ | 0.930 | 0.930 | 0.930 | 0.931 | | 0.931 | | 0.931 | | 0.931 | 0.931 | | 0.931 | | 0.931 | | 0.931 | | |
| *p ≤ 0.050. |  |  |  |  | |  | |  | |  |  | |  | |  | |  | | |
